# A Novel PQC-Based Image Encryption Scheme Using Seismic Wave Permutation

**DOI:** 10.3390/e28070800

**Published:** 2026-07-14

**Authors:** Cemile İnce

**Affiliations:** Distance Education Research and Application Center, Malatya 44280, Türkiye; cemile.ince@inonu.edu.tr

**Keywords:** post-quantum cryptography, ML-KEM, image encryption IND-CPA security, HKDF-SHA256, seismic wave permutation, nonce-based key derivation

## Abstract

Image encryption schemes based on chaotic maps offer strong statistical properties but are vulnerable to quantum attacks, and their integration with post-quantum cryptography has not been sufficiently explored. This paper presents a post-quantum secure image encryption framework integrating ML-KEM (FIPS 203), standardized by NIST in 2024, with a two-dimensional Sinh-Logistic chaotic map, HKDF-SHA256 nonce-based key derivation, feedback diffusion, and a novel Seismic Wave Permutation (SWP). The scheme derives channel-specific encryption keys from ML-KEM shared secrets using random, channel-specific nonces via HKDF-SHA256, ensuring plaintext independence and avoiding metadata-based leakage. The proposed SWP effectively breaks spatial correlations by displacing pixels according to a chaotic SWP model. RGB images are processed with independent ML-KEM encapsulation and HKDF-derived key material per channel, enabling multi-channel encryption without cross-channel leakage. Experiments on 512 × 512 test images have demonstrated Shannon entropy exceeding 7.999 bits per pixel across all channels, NPCR of at least 99.59%, UACI between 33.41% and 33.53%, and near-zero pixel correlations, further validated across 14 standard SIPI test images. An IND-CPA game simulation using four independent distinguishers, including a learned classifier trained via chosen-plaintext oracle access, over 5000 rounds per image, showed a maximum adversary advantage of 0.0186, consistent with random prediction. ML-KEM encapsulation contributes between 3.9% (ML-KEM-512) and 8.0% (ML-KEM-1024) of total encryption latency at 512 × 512 resolution, remaining a minority cost across all security levels while keeping the total encryption time within a narrow 227–258 ms range. The proposed architecture bridges standardized post-quantum cryptography with chaos-based image security for privacy-preserving image transmission.

## 1. Introduction

The rapid proliferation of digital image communication in fields such as healthcare [1,2,3], military surveillance [4,5,6], cloud storage [7,8], and social media platforms [9,10] has made the secure transmission and storage of image data even more critical. Since images often contain sensitive personal, corporate, or operational information, robust, efficient, and implementable image-encryption methods are needed to protect this data from unauthorized access.

Chaotic maps are widely used in the image-encryption literature due to their properties such as sensitivity to initial conditions, ergodicity, and pseudo-randomness [11]. These methods generally aim to disrupt the positional relationship between pixels and to propagate small changes in pixel values to spread across the encrypted image. To this end, these stages, consistent with Shannon’s principles of confusion and diffusion, are fundamental components of image-encryption designs [12]. However, generating strong histograms, high entropy, low correlation, and differential attack results alone is not sufficient for real-world use. The long-term security of an image-encryption system depends not only on its internal encryption structure but also on the security of the key generation and key sharing mechanisms used. Another important aspect, cryptographic strength at the algorithmic level, is necessary but not sufficient for real-world deployment [13]. Therefore, although algorithm-level cryptographic robustness is necessary for real-world deployment, it is not sufficient on its own [13]; system-level components such as the reliability of the random number generators used [14,15], cryptanalysis testing tools and evaluation approaches, randomness analyses, proactive attack-prevention mechanisms, and conventional intrusion detection systems should also be considered as part of a secure encryption architecture.

Quantum computing necessitates a reassessment of traditional cryptographic assumptions. While common cryptographic systems like AES, RSA, and ECC are considered secure under classical attack models, quantum algorithms threaten these systems. RSA relies on the difficulty of factoring large integers, while ECC relies on the computational difficulty of the elliptic curve discrete logarithm problem. The Shor algorithm [16] directly threatens systems like RSA and ECC because it can solve the fundamental mathematical problems on which such public-key systems are based in polynomial time. Furthermore, although the Grover algorithm does not directly break symmetric encryption algorithms like AES, it accelerates the brute-force search for the secret key using quantum computing. As a result of this acceleration, the effective security level provided by, for example, a 128-bit key can be reduced to approximately 64 bits [17]. Therefore, while the Grover algorithm does not completely eliminate the key space, it reduces the number of attempts required to find the correct key by the square root.

When the Shor and Grover algorithms are taken together, it seems that both public-key cryptography systems and symmetric encryption algorithms need to be redesigned or strengthened in the post-quantum era. Especially in image-encryption systems, simply obtaining statistically strong results is not sufficient. Supporting these systems with a quantum-resistant key management mechanism is also of great importance. Therefore, integrating post-quantum cryptography standards with image-encryption structures emerges as an important research direction.

As part of the standardization efforts in the field of post-quantum cryptography, the National Institute of Standards and Technology (NIST) published the FIPS 203 standard in August 2024 and standardized the Module Lattice-Based Key Encapsulation Mechanism (ML-KEM), previously known as CRYSTALS-Kyber [18]. ML-KEM is an IND-CCA2 secure key encapsulation mechanism based on the Module Learning with Errors (MLWE) problem and is considered resistant to both classical and quantum attacks [19,20]. Its offers three different safety levels with variants, which are ML-KEM-512, ML-KEM-768 and ML-KEM-1024, and this makes it possible to adapt this mechanism to applications with different safety requirements [18]. In this respect, ML-KEM provides a strong foundation for post-quantum secure key generation and key sharing in image-encryption systems.

In the proposed scheme, ML-KEM was selected as the key encapsulation mechanism (KEM). Following an extensive global evaluation process, this algorithm stands as the only KEM standardized by NIST as the primary post-quantum standard (FIPS 203). The security infrastructure of ML-KEM relies on the Module Learning with Errors (MLWE) problem, which is currently intractable for both classical and quantum computers. Compared to alternative post-quantum mechanisms in the literature, ML-KEM offers more compact public key and ciphertext sizes, alongside lower computational overhead. This computational efficiency is of critical importance for the proposed scheme, given that three independent encapsulation processes are executed independently for the color channels of each RGB image. Consequently, its standardized status, robust theoretical security foundation, and practical performance advantages justify its selection over alternative post-quantum constructions.

In the current image-encryption literature, chaotic confusion–diffusion structures, statistical security analyses, quantum resistive key management, and attack distinguishability are mostly handled as separate problems. Many studies evaluate the image-encryption success based on metrics such as histogram uniformity, high entropy, low pixel correlation, ideal NPCR, and UACI values. However, it does not address the post-quantum security of the key generation mechanism used within the same design. On the other hand, although post-quantum key encapsulation mechanisms offer a cryptographically strong foundation, how to integrate them into chaos-based image-encryption schemes, how to support the decryption accuracy of auxiliary data, and how to evaluate the system’s distinguishability behavior under selected plaintext attacks have been studied in limited form in the literature.

In order to address this research gap, an end-to-end encryption scheme that integrates the ML-KEM-based key generation mechanism with a chaos-based image encryption is presented in this study. The proposed structure combines a new pixel scrambling mechanism called Seismic Wave Permutation (SWP) with a feedback XOR-modular diffusion stage. SWP arranges pixel positions using a time-of-arrival model that combines a chaotic perturbation sequence with a geometric distance term computed from a secret, key-derived epicenter, inspired by the seismic propagation pattern; both components must be known to reconstruct the permutation, coupling the spatial rearrangement to two independently derived secret parameters. In the diffusion stage, each encrypted pixel is generated depending on the current plaintext image pixel, two different key flow values, and the previous encrypted pixel. Thus, the proposed architecture forms an integrated encryption structure that provides both spatial scrambling and pixel value diffusion. In parallel with the research question mentioned, it was investigated how ML-KEM can be integrated into a chaos-based image-encryption line and how this integration can support both accurate decryption and strong security results. In this context, it is aimed that the proposed system maintains internal consistency in terms of algorithm definition, supports sensitive decryption through transmitted data, produces strong results in statistical security tests, and provides a low distinguishability advantage under selected plaintext attacks.

The remainder of this article is structured as follows: Section 2 reviews related work on chaos-based image encryption, post-quantum cryptography, and IND-CPA analysis. Section 3 details the proposed scheme, including system overview, key derivation, diffusion phase, novel SWP permutation, and decryption. Section 4 describes the experimental setup. Section 5 reports the cryptanalysis results, visual outputs, timing measures, and selected plaintext discriminability experiments. Section 6 discusses the main findings and limitations. Section 7 concludes this article.

## 2. Related Work

Research in the field of image encryption has evolved from conventional text encryption algorithms toward specialized designs tailored to image-specific characteristics, such as large data volumes and high pixel correlation [21]. Although early works directly applied traditional block cipher methods to images, subsequent works revealed that the structural properties of images, namely high redundancy and pixel correlation, render confusion–diffusion architectures significantly more efficient. Particularly in recent years, chaos-based methods have emerged as a dominant approach in the literature, offering low computational costs, strong permutation capabilities, and fast nonlinear transformations [22]. Recent studies continue to expand this broad field through encryption methods based on Substitution-Permutation Network (SPN) architectures [23], compressed image encryption [24], image protection for optical communications and high-altitude platform links [25,26], memristive and hyperchaotic systems [27,28,29], multi-image designs [29,30,31], and lightweight structures for resource-constrained environments [32,33].

The expansion of the chaos-based image-encryption literature in recent years has led to new searches for optimizing confusion and diffusion properties. In this regard, research focuses on DNA-based processes, dynamic coding rules, and nonlinear substitution mechanisms [34,35,36]. Parallel to this, some studies utilize memristive, fractional-order, or hyper-chaotic systems to increase the state-space diversity of chaotic maps, thus producing more complex, unpredictable, and non-periodic sequences [37,38,39]. On the other hand, the literature shows that in real-world applications, cryptographic systems should be evaluated not only by their security levels but also by practical applicability criteria such as encryption–decryption speed and hardware cost [32,39]. When these studies are considered holistically, it is seen that while the image-encryption literature offers a rich variety in terms of pixel-level operations, it remains weak in terms of systematic key management and mathematical security proofs. Furthermore, the quantum threats revealed by the Shor and Grover algorithms have made it imperative for long-term security to also take these dynamics into account [16,40]. As part of the transition to the post-quantum era, NIST standardized the ML-KEM (FIPS 203) algorithm, providing a post-quantum key encapsulation mechanism for practical deployments. Current studies focus on integrating these mechanisms into authenticated session protocols [41,42,43].

Image-encryption algorithms fundamentally consist of confusion and diffusion steps. Using different approaches, the pixels of the image are permuted to each other, aiming to disrupt the image-specific neighbor pixel correlations and strengthen the encryption process [44]. In the literature, various pixel permutation methods have been proposed, such as ZigZag and its variants, Spiral, Corner, and Knit Scrambling [45,46,47,48]. Existing permutation algorithms exhibit distinct trade-offs. ZigZag and Spiral, despite having multiple variants, are relatively simple and consequently leave a relatively high number of unchanged pixels. The Corner algorithm, despite its deterministic structure, achieves significantly fewer unchanged pixels compared to alternatives (0–2 pixels in test images). Knit Scrambling has been reported to achieve zero unchanged pixels; however, its dedicated framework structure makes it difficult to integrate into other encryption schemes [49]. The proposed SWP algorithm addresses both challenges: it produces high permutation quality comparable to alternatives while maintaining compatibility with diverse encryption architectures.

Recent literature surveys evaluate image-encryption methods based on chosen-plaintext (CPA) and chosen-ciphertext (CCA) attacks [50]. Despite this, many studies still restrict security assessments to statistical metrics such as entropy, NPCR, UACI, and pixel correlation [11,13,33]. While useful, these metrics provide no assurance regarding cryptographic indistinguishability or resistance to active attacks. Indeed, cryptanalysis works have demonstrated that certain schemes can be broken under CPA or CCA, even with strong statistical outputs [51,52]. Moreover, authentication mechanisms in the literature are typically presented as standalone components appended after encryption rather than being integrated into the process [53]. Consequently, passing statistical tests does not guarantee CPA security; such security must be deliberately embedded into the system during the design phase [54].

Table 1 summarizes representative image cipher studies, emphasizing the confusion–diffusion strategy, key management, and computational profile. This table shows that recent studies are rich in terms of image processing and strong in terms of statistical test results. However, it shows that standardized post-quantum key generation, clear and explicit algorithm definition, and analyses based on formal security models are still not widespread.

The literature also confirms two axioms for the current work:

**Observation 1:** Strong statistical character alone does not provide cryptographic indistinguishability [50,52].

**Observation 2:** A future-oriented image cipher architecture should not exclude key generation from post-quantum transition [42,55]. These observations lead to two research gaps:

**Research Gap 1—Architecture:** Recent chaotic image ciphers rarely incorporate a standardized post-quantum KEM into the encryption pipeline [13,42].

**Research Gap 2—Evaluation:** Recent studies rarely correlate image-encryption results with explicit CPA- or CCA-style reasoning and generally dwell on statistical indicators [11,50,52,53].

**Table 1:** Comparison of image-encryption schemes in terms of confusion–diffusion strategies and key characteristics.

Ref	Image Type	Confusion	Diffusion	Key Space	Time Complexity	Key Mechanism
[27]	Color	Memristor hyperchaotic + Trivium	DNA ops + lightweight 3DES layer	$2^{512}$	$O(10\cdot N^{2})$	Chaotic initial cond.
[28]	Color	2D-SEMS map	Hexadecimal perm. + 2D cumulative diff.	$2^{398}$	$O(4\cdot N^{2})$	Chaotic key gen
[29]	Color	10D+8D hyperchaotic + CNN S-box	Multi-layer S-box XOR FPGA parallel	$2^{3454}$	$O(8\cdot N^{2})$	BBS S-box + SHA
[30]	Color (multi)	4D Chen hyperchaotic + 6 novel S-boxes	Parallel RGB cube XOR + DNA-like	$2^{4624}$	$O(6\cdot N^{2})$	Mersenne Twister
[31]	Multi (sat.)	Hyperchaotic SVD + Hill cipher	RC5 counter mode + custom S-box	$2^{512}$	$O(12\cdot N^{2})$	BBS PRNG + SHA
[34]	Color	3D hyperchaotic + dynamic RNA/DNA	RNA + DNA XOR dual diffusion	–	–	Chaotic initial vals
[37]	Color	5D PD5H hyperchaotic	Pixel-block internal + flow joint diff.	$2^{256}$	–	Plaintext SHA hash
[38]	Color (typhoon)	OTWFM model	Clockwise vortex + wind-field diff.	$2^{299}$	–	Chaotic params
[56]	Color (multi)	2D-SLEM chaotic map	Block space jump + sliding queue XOR	$2^{604}$	$O(6\cdot N^{2})$	SHA-512
[26]	Color	5D hyperchaotic system + DNA coding	DNA XOR arithmetic + chaotic diff.	–	$O(8\cdot N^{2})$	SHA-256
Prop.	RGB	SWP + Sinh-logistic chaos	Feedback XOR + HKDF-SHA256	$2^{256}$	$O(N^{2}\log N)$	ML-KEM-512 (PQC)

Note: Entries include single-color and multi-image schemes; key space/complexity values are not directly comparable across image types.

In other words, there are many ideas of strong image-dependent layers and many strong statistical results in the literature, but very few studies link post-quantum key generation, precise algorithm definition, and hostile evaluation to a single consistent design. These results give rise to the following research questions:

Research question 1: Can a chaos-based image-encryption scheme be constructed around ML-KEM as the key generation layer?

Research question 2: Can such a scheme remain operationally open, support precise decryption via transmitted auxiliary data, and still offer strong image security measures along with a meaningful selected plaintext evaluation?

This study addresses these limitations by combining ML-KEM-based key generation with HKDF-based parameter derivation, feedback diffusion, and SWP permutation within a single image-encryption scheme. This study also incorporates statistical testing, timing analysis, and selected plaintext evaluation within the same framework. In this sense, the contribution of this study is not only a novel hybrid design but also a clearer bridge between standardized post-quantum key generation and practical chaos-based image encryption. Therefore, the contribution is both methodological and architectural.

In light of all the literature reviewed, the main contributions of this study can be summarized as follows:

- An ML-KEM-integrated chaos-based image-encryption framework is proposed to support post-quantum secure key establishment.
- A new pixel-level permutation method, named Seismic Wave Permutation (SWP), is introduced, coupling a chaotic perturbation sequence with a key-derived geometric epicenter so that reconstructing the permutation requires two independently derived secret components.
- The proposed framework combines SWP-based permutation with a feedback XOR-modular diffusion stage to form a complete confusion–diffusion encryption pipeline.
- The integration of an NIST-standardized post-quantum key encapsulation mechanism (ML-KEM) with nonce-based HKDF-SHA256 key derivation for chaos-based image-encryption parameters is proposed, ensuring plaintext-independent, IND-CPA-secure key material under a post-quantum threat model, empirically validated through extensive adversarial simulation.
- The scheme is evaluated using statistical security metrics, timing analysis, and an explicit IND-CPA adversarial simulation, demonstrating that statistical metrics alone are insufficient to establish cryptographic indistinguishability and that explicit adversarial modeling is necessary in image-encryption research.
- This study bridges post-quantum key management and chaos-based image encryption within a single coherent design.

## 3. Proposed Encryption Scheme

**Figure 1:**
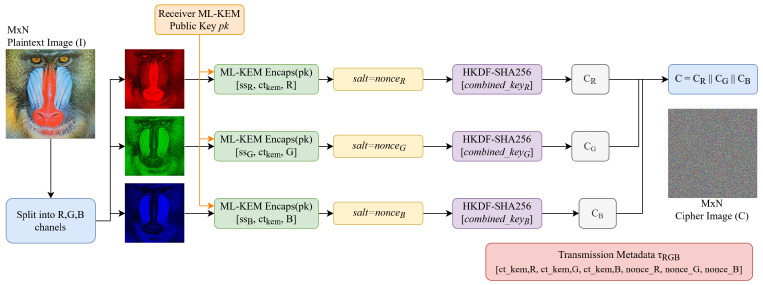
Proposed encryption scheme flowchart.

The proposed method consists of two main stages: key generation and image encryption. In the first stage, channel-specific shared secrets are independently established for the R, G, and B channels using ML-KEM encapsulation. These channel-specific shared secrets are then extended using the HKDF-SHA256 method to securely generate the sub-parameters and session-specific keys needed in the encryption process. In the second image-encryption stage, these derived parameters are used. In this step, pixel values are first scrambled using feedback XOR-modular diffusion, and then pixel positions are rearranged using the novel SWP algorithm. Applying these two processes together provides a strong encryption structure by altering both the pixel value and the position (spatial arrangement) of the pixels. The graphical abstract of the proposed method is presented in Figure 1.

**Figure 2:**
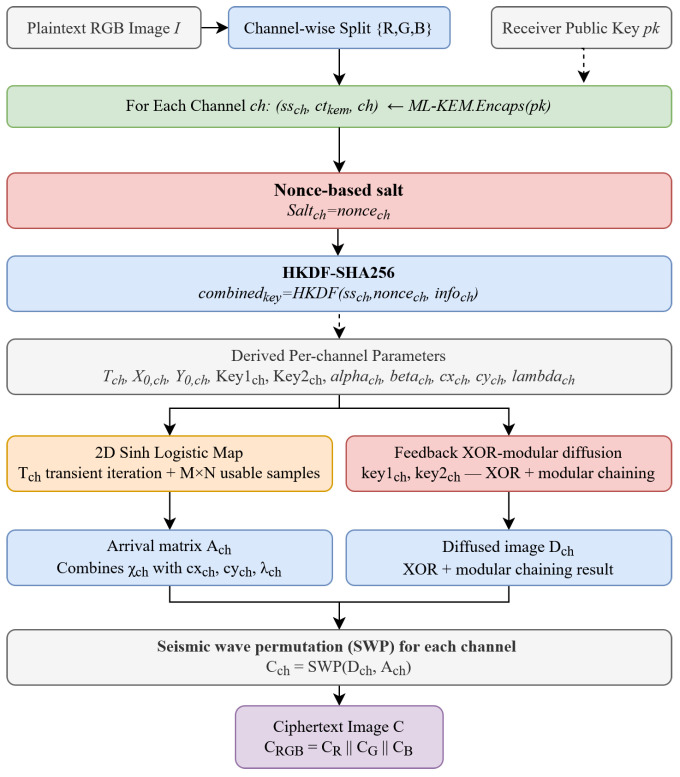
Proposed encryption scheme architecture overview.

The proposed scheme takes two inputs: an RGB plaintext image *I* of dimensions M×N and the receiver’s ML-KEM public key pk. For an RGB image, the ML-KEM encapsulation process is performed independently for each color channel. For each channel ch∈{R,G,B}, ML-KEM encapsulation creates a channel-specific shared secret key $ss_{ch}$ and KEM ciphertext $ct_{\mathrm{kem},ch}$. In the image-encryption layer, the channel-specific combined key is derived from the shared secret key $ss_{ch}$ using HKDF-SHA256 with a random, channel-specific nonce $nonce_{ch}$ as the salt. This design supports plaintext independence and empirically exhibits near-random adversarial success in the IND-CPA-style experiment. The exact derivation process is explained in detail in Section 3.1. This *combined_key_ch_* is then used as input to HKDF calls separated by domain name (each initialized with a different *info* string), and separate parameters are generated for different tasks. These parameters include the number of temporary iterations $T_{ch}$, the initial and control values of the chaotic map, the key flows to be used during the diffusion phase, and the control parameters required for SWP. The 2-dimensional Sinh-Logistic map is initialized and run for $T_{ch}$ transient iterations, which are discarded to reduce the direct impact of the initial conditions, then M×N additional iterations are performed to create the usable output sequence for the corresponding channel. In the first stage, feedback XOR modular diffusion is applied to the channel pixel array, and each pixel value is made dependent on the previous ciphertext value (feedback chain). In the final stage, the scattered pixels are repositioned using SWP. Thus, both pixel values and pixel positions are changed together. In other words, while the first stage hides the pixel values, the second stage hides the spatial information of the image. The SWP algorithm is applied independently to each color channel and is designed to ensure that no pixel remains in its original position, so that the distortion of spatial correlations is maximized. The complete block diagram of the proposed encryption scheme is shown in Figure 2.

The scheme produces two components as output. Namely, the encrypted image *C* and the accompanying compact transmission block τ. In practical use, the τ block carries the channel-specific KEM ciphertexts $ct_{\mathrm{kem},R}$, $ct_{\mathrm{kem},G}$, and $ct_{\mathrm{kem},B}$, together with the auxiliary information necessary for the receiver to regenerate the same parameters during decryption. Specifically, the transmitted metadata includes a random channel-specific nonce, denoted $nonce_{ch}$, used as the HKDF salt, which supports plaintext independence and avoids metadata leakage from the plaintext. In this construction, *T* (the transient iteration count) is derived solely from the combined key and requires no plaintext-dependent auxiliary data.

Accordingly, the transmitted metadata is defined as

(1) $$\tau_{RGB}=\left\{ct_{\mathrm{kem},R},ct_{\mathrm{kem},G},ct_{\mathrm{kem},B},nonce_{R},nonce_{G},nonce_{B}\right\}$$

On the receiver side, each channel-specific KEM ciphertext is decapsulated independently to recover the corresponding shared secret:

(2) $$ss_{ch}\leftarrow \mathrm{ML}\text{-}\mathrm{KEM}.\mathrm{Decaps}\left(sk,ct_{\mathrm{kem},ch}\right),\;ch\in \left\{R,G,B\right\}.$$

The channel-specific combined key is then reconstructed using the transmitted channel-specific nonce, $nonce_{ch}$, as the HKDF salt.

Chaotic-map parameters, diffusion key streams, and SWP parameters are then regenerated from $combined\text{\_}key_{ch}$ for each channel. Finally, reverse SWP and reverse diffusion are applied independently to each channel, and the recovered channels are concatenated to reconstruct the original RGB image *I*.

In this structure, the security of the key generation layer directly relies on the security assumptions of ML-KEM. The image-encryption layer, however, was evaluated experimentally in this study not through formal proof, but through statistical analyses and selected plaintext distinguishability experiments.

### 3.1. Post-Quantum Key Encapsulation and Nonce-Based Key Derivation

The key generation phase is responsible for securely generating the encryption parameters that will control the image cipher. Instead of relying on classical key exchange protocols such as ECDH, which are vulnerable to quantum attacks via the Shor algorithm [57], the proposed scheme uses ML-KEM, a key encapsulation mechanism whose security is based on the difficulty of the MLWE problem [19], which is considered intractable for both classical and quantum adversaries [58]. The encapsulation process takes the receiver’s ML-KEM public key pk as input and internally samples fresh encapsulation randomness. It produces two outputs simultaneously, as given in Equation (3).

(3) $$\left(ss_{ch},ct_{\mathrm{kem},ch}\right)\leftarrow \mathrm{ML}\text{-}\mathrm{KEM}.\mathrm{Encaps}\left(pk\right),\;ch\in \left\{R,G,B\right\}.$$

where $ct_{\mathrm{kem},ch}$ is the channel-specific KEM ciphertext transmitted alongside the encrypted image, and $ss_{ch}$ is the 32-byte channel-specific shared secret used only for the corresponding color channel. On the recipient’s side, the shared secret is recovered from $ct_{kem}$ using the private key sk, as given in Equation (4).

(4) $$ss_{ch}\leftarrow \mathrm{ML}\text{-}\mathrm{KEM}.\mathrm{Decaps}\left(sk,ct_{\mathrm{kem},ch}\right),\;ch\in \left\{R,G,B\right\}.$$

Crucially, the security guarantee of ML-KEM ensures that no classical or quantum adversary can recover $ss_{ch}$ from $ct_{\mathrm{kem},ch}$ without knowledge of sk. In a correct ML-KEM encapsulation, each encryption session uses fresh encapsulation randomness and produces a fresh shared secret $ss_{ch}$, even under reuse of the same recipient key pair. Therefore, key reuse is not caused by reusing the recipient’s pk/sk pair, but could arise from an implementation error, deterministic encapsulation randomness, or accidental reuse of the same derived image-encryption key. To avoid direct dependence on raw KEM output, the proposed scheme derives all image-layer parameters from $ss_{ch}$ using HKDF-SHA256 with a random, channel-specific nonce.

The role of ML-KEM in the proposed architecture is limited to establishing the channel-specific shared secrets $ss_{ch}$ between the sender and the receiver. The image-encryption layer, however, requires several deterministic parameters, including chaotic-map initial values, diffusion key streams, transient iteration counts, and SWP parameters. Therefore, $ss_{ch}$ is not used directly in the image-encryption process. Instead, it is first transformed into an intermediate key, denoted as $combined\text{\_}key_{ch}$, by using HKDF-SHA256 with a random, channel-specific nonce as the salt.

Each channel-specific combined key is derived using a channel-specific random nonce as the HKDF salt:

(5) $$nonce_{ch}\leftarrow {\left\{0,1\right\}}^{256},$$

(6) $$\begin{array}{ccc} combined\text{\_}key_{ch}\; & = & \mathrm{HKDF}\text{-}\mathrm{SHA256}(\\ \; & \; & \left.\;ss_{ch},salt=nonce_{ch},info=“\mathrm{IND}\text{-}\mathrm{CPA}\text{-}\mathrm{Key}\text{-}”\|ch\right) \end{array}$$

This construction ensures that encrypting the same image twice produces completely different ciphertexts, as a result, the construction supports plaintext independence and empirically exhibits near-random adversarial success in the IND-CPA-style experiment.

After the combined key is obtained, the parameters to be used in the image layer are derived in an orderly manner.

### 3.2. HKDF-SHA256 Expansion

HKDF is a standard key derivation function defined in RFC 5869 [59]. It uses a two-stage structure to generate secure and fixed-length outputs from raw key data: extraction, which converts input keying material into a pseudo-random key (PRK) via HMAC, and expansion, which generates output keys (OKM) of the desired length from this PRK.

As described in Section 3.1, $combined\text{\_}key_{ch}$ is itself the output of an HKDF-SHA256 extract-and-expand operation over $ss_{ch}$, and is therefore already uniformly distributed. Consequently, $combined\text{\_}key_{ch}$ is used directly as the PRK for a further HKDF-Expand step, without performing a fresh extraction. The two diffusion key streams, key1 and key2, each of length M×N bytes, are derived as given in Equations (7) and (8).

(7) $$key1_{ch}=\mathrm{HKDF}\text{-}\mathrm{Expand}\left(combined\text{\_}key_{ch},“\mathrm{keystream}\text{-}1\text{-}”\|ch,M\times N\right)$$

(8) $$key2_{ch}=\mathrm{HKDF}\text{-}\mathrm{Expand}\left(combined\text{\_}key_{ch},“\mathrm{keystream}\text{-}2\text{-}”\|ch,M\times N\right)$$

### 3.3. Chaotic Map: 2D Sinh-Logistic System

The proposed scheme utilizes a two-dimensional Sinh-Logistic chaotic map to generate the pseudo-random perturbation sequence that controls the Seismic Wave Permutation (SWP) stage. The working principle of this map is described by the update equations given in Equations (9) and (10).

(9) $$x\left(n+1\right)=|\alpha \cdot \sinh\left(x_{n}\right)\cdot \left(1-x_{n}\right)|\;\mathrm{mod}\;1$$

(10) $$y\left(n+1\right)=|\beta \cdot y_{n}\cdot \left(1-y_{n}\right)+x\left(n+1\right)|\;\mathrm{mod}\;1$$

**Figure 3:**
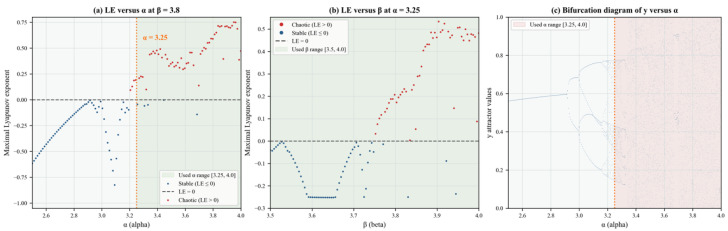
Lyapunov exponent analysis and bifurcation diagram of the two-dimensional Sinh-Logistic chaotic map.

The map, initialized with the derived initial conditions ($x_{0}$, $y_{0}$), is run through a total of $T_{ch}+M\times N$ steps, where $T_{ch}$ represents the channel-specific transient iteration count. Figure 3 shows the parameter range in which the 2D Sinh-Logistic map behaves chaotically; α and β are accordingly restricted to this verified range during parameter derivation (Section 3.1). The first *T* steps are discarded because they represent the disordered portion of the system before it reaches stable chaotic behavior, thus preventing the addition of unnecessary noise to the output sequence. At each of the remaining M×N steps, the two state variables are combined as given in Equation (11), producing the chaotic perturbation matrix $\chi \in R^{M\times N}$.

(11) $$\chi \left(i,j\right)=\left(x_{n}+y_{n}\right)\;\mathrm{mod}\;1$$

Combining both state variables, rather than using either in isolation, suppresses the short-range correlation that the one-directionally coupled component (*x*, which does not receive feedback from *y*) can otherwise exhibit. This perturbation matrix is then converted into a time-of-arrival matrix of size $A\in R^{M\times N}$ (Section 3.5), which assigns a sequence value to each pixel, determining the order in which the pixels are rearranged.

#### Finite Precision Considerations

While chaotic maps in continuous mathematics exhibit infinite precision properties, numerically they have IEEE 754 floating-point arithmetic constraints. Finite precision can lead to dynamic distortions after extensive iterations [11]. The proposed implementation employs IEEE 754 double precision (64-bit) arithmetic, providing approximately 15–17 decimal digits of accuracy. For a 512×512 RGB image, the chaotic map executes approximately 786,432 production iterations (262,144 pixels × 3 channels), plus a small per-channel transient of T∈[50,255] iterations. This iteration count remains well below the degradation thresholds reported in the literature, where double-precision chaotic systems typically maintain randomness properties for $10^{6}$–$10^{7}$ iterations before precision-induced periodicity emerges [47]. The practical impact of finite precision on cryptographic security is evaluated through statistical metrics and IND-CPA simulation in Section 5 and Section 6.

### 3.4. Feedback XOR-Modular Diffusion Phase

Since the same diffusion rule is applied independently to each color channel, the channel index ch is omitted in this subsection for notational simplicity. The diffusion stage works on the flattened plaintext pixel array $P=[p_{0},p_{1},\ldots ,p_{MN-1}]$ of a single channel, generating the corresponding diffused output array $D=[d_{0},d_{1},\ldots ,d_{MN-1}]$. As described in Section 3.2, two independent key streams ($key_{1}$ and $key_{2}$), each MN bytes long, are obtained using HKDF-SHA256.

In this stage, the diffusion process is performed step-by-step in a one-dimensional chaining structure, where each pixel is also dependent on the results preceding it as presented in Equations (12) and (13).

(12) $$d_{0}=\left(p_{0}⊕key1_{0}+key2_{0}\right)\;\mathrm{mod}\;256$$

(13) $$d_{i}=\left(p_{i}⊕key1_{i}+key2_{i}+d_{i-1}\right)\;\mathrm{mod}\;256,\;i=1,2,\ldots ,M\times N-1$$

where all arithmetic is performed modulo 256. The two key streams play complementary roles. $key_{1}$ creates bit-level confusion via XOR, while $key_{2}$ creates arithmetic spread via modular addition. Together, they ensure that each output byte is simultaneously linked to a plaintext pixel, both key stream values, and a chained ciphertext state.

The feedback term $d_{i-1}$ in Equation (13) creates a chaining dependency across the pixel array. Therefore, a change in a single pixel in the plaintext first affects the $d_{i-1}$ value, and then this effect propagates to all subsequent pixels. This propagation effect (avalanche effect) is necessary to obtain high NPCR values and is experimentally demonstrated in Section 5.

For each channel, using the same channel-specific key streams, the decryption inverse is given by the following equations:

(14) $$p_{0}=\left(d_{0}-key2_{0}\right)\;\mathrm{mod}\;256⊕key1_{0}$$

(15) $$p_{i}=\left(d_{i}-key2_{i}-d_{i-1}\right)\;\mathrm{mod}\;256⊕key1_{i},\;i=1,2,\ldots ,M\times N-1$$

which is also consistent with the decryption implementation in the proposed scheme.

### 3.5. Proposed SWP

**Figure 4:**
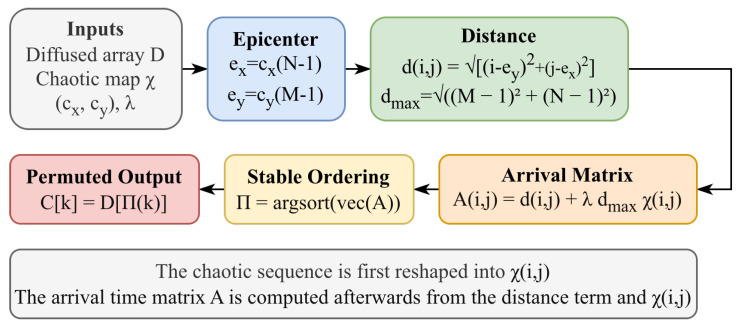
Operational view of SWP.

SWP is a spatial reordering step implemented after the feedback XOR-modular diffusion step and developed for this study. SWP does not alter already-spread pixel densities. Instead, it changes their spatial positions according to a time-of-arrival model inspired by wave propagation from a virtual epicenter. In the current implementation, SWP is driven by three derived parameters: epicenter coordinates ($c_{x}$, $c_{y}$), the decay coefficient λ, and a chaotic perturbation matrix χ derived from a 2D Sinh-Logistic map (Section 3.3). The epicenter coordinates and decay coefficient are derived directly from combined_key via HKDF-SHA256 with a dedicated domain-separation string, independently of the chaotic map: $c_{x}\in \left\{0,\ldots ,N-1\right\}$, $c_{y}\in \left\{0,\ldots ,M-1\right\}$, and $\lambda =0.1+0.4\cdot u_{\lambda }$ for $u_{\lambda }\in \left[0,1\right)$, yielding λ∈[0.1,0.5]. This range ensures that neither the Euclidean distance term nor the chaotic perturbation term dominates, maintaining a balanced contribution from both components in the arrival matrix computation. Let $D\in {\left\{0,\ldots ,255\right\}}^{M\times N}$ represent the spread image. For each pixel position (*i*, *j*), the Euclidean distance from the epicenter ($c_{x}$, $c_{y}$) is calculated. This geometric term defines the basic arrival structure, while the chaotic term disrupts this structure, preventing a simple radial ordering. Complete steps are presented in Equations (16)–(19). Figure 4 presents an operational view of this process.

(16) $$d\left(i,j\right)=\sqrt{{\left(i-c_{y}\right)}^{2}+{\left(j-c_{x}\right)}^{2}}$$

(17) $$d_{max}=\sqrt{{\left(M-1\right)}^{2}+{\left(N-1\right)}^{2}}$$

(18) $$A\left(i,j\right)=d\left(i,j\right)+\lambda d_{max}\chi \left(i,j\right)$$

(19) Π=argsort(vec(A)),C[k]=D[Π(k)].

**Figure 5:**
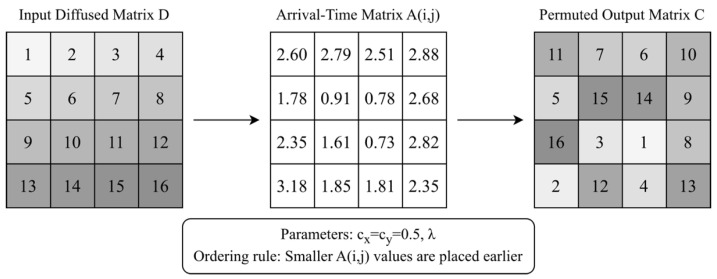
Compact 4 × 4 SWP example with a representative image-like block.

SWP remains fully invertible and therefore suitable for lossless image recovery within the proposed encryption pipeline. Figure 5 shows a compact 4 × 4 example illustrating this transformation on a representative image-like block.

### 3.6. Decryption Algorithm

Decryption is the exact inverse of encryption and requires the ML-KEM secret key sk and the transmitted metadata block. The metadata block is

(20) $$\begin{array}{ccc} \tau_{RGB}\; & = & \{ct_{\mathrm{kem},R},ct_{\mathrm{kem},G},ct_{\mathrm{kem},B},nonce_{R},nonce_{G},nonce_{B}\}. \end{array}$$

For each channel ch∈{R,G,B}, the recipient decapsulates the corresponding KEM ciphertext as

(21) $$ss_{ch}\leftarrow \mathrm{ML}\text{-}\mathrm{KEM}.\mathrm{Decaps}\left(sk,ct_{\mathrm{kem},ch}\right).$$

For each channel ch∈{R,G,B}, the recipient decapsulates $ct_{\mathrm{kem},ch}$ to recover $ss_{ch}$.

(22) $$\begin{array}{ccc} combined\text{\_}key_{ch}\; & = & \mathrm{HKDF}\text{-}\mathrm{SHA}256(\\ \; & \; & \left.ss_{ch},\;salt=nonce_{ch},\;info=“\mathrm{IND}\text{-}\mathrm{CPA}\text{-}\mathrm{Key}”\|ch\right) \end{array}$$

All channel-specific encryption parameters $T_{ch}$, $\left(x_{0,ch},y_{0,ch},\alpha_{ch},\beta_{ch}\right)$, $\left(key1_{ch},key2_{ch}\right)$, and $\left(c_{x,ch},c_{y,ch},\lambda_{ch}\right)$ are retrieved via HKDF-SHA256 using the same domain-separated info strings as in encryption. The chaotic perturbation matrix χ is regenerated identically by re-running the 2D Sinh-Logistic map for the same $T_{ch}+M\times N$ steps. The arrival time matrix *A* is reconstructed from these values, and the inverse SWP permutation where Π maps ciphertext indices to diffused-array indices; the inverse permutation is computed accordingly: $\Pi^{-1}=\mathrm{argsort}\left(\Pi \right)$ is applied to recover the diffused array *D* from the ciphertext *C*. The inverse diffusion then recovers the plaintext pixel array using Equations (14) and (15).

## 4. Experimental Setup

### 4.1. Test Images

Three standard RGB color images from the USC-SIPI Image Database [60], Peppers, Baboon, and House, were used for evaluation. The images are used at their native 512×512×3 resolution as provided in the database, without downsampling.

### 4.2. Implementation

The proposed scheme is implemented in *Python 3.10* using the *kyber-py* library for ML-KEM operations, a pure-Python implementation providing ML-KEM-512, ML-KEM-768, and ML-KEM-1024 in accordance with FIPS 203. Chaotic-map iterations and pixel-level operations are implemented using *NumPy* for efficient array processing. Key derivation is performed via a custom HKDF-SHA256 implementation based on RFC 5869 [59] built on Python’s standard *hmac* and *hashlib* modules, and plaintext hashing is performed using the standard *hashlib* module. All experiments are conducted on an x86-64 Linux system. Timing measurements are averaged over 60 independent runs per configuration to reduce variance. The implementation is purely software-based without hardware acceleration, representing a conservative performance baseline.

### 4.3. RGB Color Image Support

RGB images are encrypted by deriving independent key material for each color channel (R, G, and B). This design provides cryptographic isolation between channels and enables parallel processing.

For an RGB image of size M×N×3, the encryption process operates as follows:

(23) $$C_{RGB}=\mathrm{Encrypt}\left(I_{R}\right)\;||\;\mathrm{Encrypt}\left(I_{G}\right)\;\left|\right|\;\mathrm{Encrypt}\left(I_{B}\right)$$

where $I_{R}$, $I_{G}$, and $I_{B}$ represent the Red, Green, and Blue channels, respectively, and || denotes concatenation. Each channel encryption uses independent HKDF-derived parameters with channel-specific info strings (“channel-R”, “channel-G”, and “channel-B”) to ensure statistical independence.

The metadata structure is extended to include per-channel parameters:

(24) $$\tau_{RGB}=\left\{ct_{\mathrm{kem},R},ct_{\mathrm{kem},G},ct_{\mathrm{kem},B},nonce_{R},nonce_{G},nonce_{B}\right\}$$

Each $nonce_{ch}$ is a random, channel-specific value used as the HKDF salt during key derivation (Section 3.1). The transient iteration count $T_{ch}$ is derived from $combined\text{\_}key_{ch}$ and therefore does not need to be transmitted. During decryption, the Red, Green, and Blue channels are decrypted independently using their own metadata and are then combined to reconstruct the original RGB image.

Experiments on three standard RGB test images (Peppers, Baboon, and House; 512×512 pixels) demonstrated that per-channel independent encryption provided strong statistical security across all color channels (NPCR ≥ 99.59%, UACI 33.4–33.5%, and entropy > 7.999 bits/pixel). Decryption fully reconstructed the original RGB image without loss of color information.

### 4.4. Evaluation Metrics

Table 2 presents the security metrics, definitions, and their ideal values for image-encryption evaluation used in this study:

## 5. Cryptanalysis Results

### 5.1. Statistical Security Metrics

Table 3 presents the statistical security metrics for all three RGB test images (Peppers, Baboon, and House) encrypted with the proposed scheme instantiated with ML-KEM-512 and scrambled with SWP algorithm. All metrics meet or closely approximate their ideal theoretical values across all color channels, confirming the strong statistical security of the proposed scheme.

**Figure 6:**
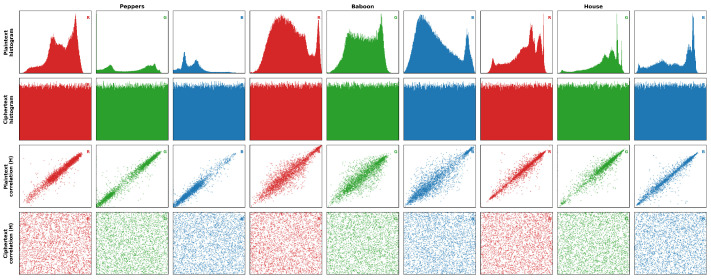
Histogram and adjacent pixel correlation analysis of original and encrypted images (Pepper, Baboon, and House) with proposed scheme.

In the encryption process, the histogram distribution of the encrypted image pixels should be distorted, and a near-uniform distribution is expected. Another characteristic of image data is that the correlation values of neighboring pixel values should be high. The results obtained from the encryption schemes should ensure that there is no statistical data leakage in the ciphertext image. The visual representation of the numerical expressions presented in Table 3 is shown in Figure 6. Figure 6 shows the histogram and correlation analysis in three directions for three different RGB test images (Peppers, Baboon, and House) used in the tests. When Table 3 and Figure 6 are examined in parallel, they confirm the high Shannon entropy obtained by transforming the structured, non-uniform histograms of the plaintext images into a uniform distribution after the encryption process. The adjacent correlation distribution plots show that the strong linear dependencies observed in the original images with correlation coefficients ranging from 0.81 to 0.98 in all directions do not show a distinct structure in the distribution plots, and the correlation coefficients approach zero in all three directions.

The ciphertext entropy values for Peppers, Baboon, and House exceed 7.997 bits/pixel across all RGB channels, remaining within 0.003 bits/pixel of the theoretical maximum of 8.0 for a uniformly distributed 8-bit random variable. The encryption effectively converts structured image data into nearly uniform ciphertext. The ciphertext histograms exhibit nearly uniform distributions across all 256 intensity levels in each color channel, in contrast to the highly non-uniform plaintext histograms which reflect the natural statistics of the test images and prevent frequency-based statistical attacks.

The NPCR values exceed 99.59% across all test images. This almost complete avalanche effect is a direct consequence of the feedback diffusion chain. A single pixel change propagates to all subsequent pixels via the chaining term $d_{i-1}$ in Equation (13), and the SWP permutation distributes this change globally across the ciphertext. The UACI values range from 33.41% to 33.53%, remaining close to the theoretical ideal of 33.4635%, indicating that the magnitude of the change is distributed almost optimally across the pixel intensity range.

The correlation of adjacent pixels in the ciphertext is close to zero in all directions across all three RGB test images, compared to the strong positive correlations in plaintext images as reported in Table 3. The SWP permutation, like other permutation methods, was developed to disrupt spatial locality. Pixels that are adjacent in the original image are deliberately distributed to distant locations in the ciphertext, following the arrival time order defined in Equation (19). This order is pseudo-random and depends on both the image content and the ML-KEM session key. Thus, it is unique to each encryption instance and cannot be reproduced without the correct key.

To further validate the generalizability of the proposed scheme beyond the three primary test images, correlation coefficients were additionally computed across 14 standard grayscale-converted SIPI test images. As shown in Table 4, plaintext correlations range from 0.73 to 0.99 across all three directions, while the corresponding ciphertext correlations remain within ±0.009 of zero for every image, with average values of 0.0000 (horizontal), 0.0013 (vertical), and 0.0012 (diagonal). This confirms that the strong decorrelation observed for the three primary test images generalizes consistently across a broader and more diverse set of natural images.

### 5.2. Computational Analysis

Table 5 shows encryption time analysis for the Peppers, Baboon, and House images. For each image, the total time is decomposed into ML-KEM time and image processing time. For each image and ML-KEM variant, the table lists the KEM-only time, the image-layer encryption time, the total encryption time, and the total decryption time. Each value is the mean of 60 runs on a 512×512×3 RGB image, with a warm-up iteration applied before measurement. Because an RGB image is encrypted as three independent channels, each with its own ML-KEM encapsulation, the reported KEM time is the sum of all three per-channel encapsulations.

Within the image-processing layer, the dominant computational costs are the chaotic-map iteration (Section 3.3) and the feedback XOR-modular diffusion phase, both implemented as sequential per-pixel loops; SWP permutation and HKDF-SHA256-based parameter derivation contribute comparatively little. Because the image-layer loop count depends only on the image dimensions (M×N×3) and not on pixel content, the small variation observed across the three test images reflects ordinary measurement variance rather than image complexity.

In contrast to the image layer, the ML-KEM contribution scales directly with the chosen security level. Averaged across the three test images, ML-KEM-512, ML-KEM-768, and ML-KEM-1024 account for approximately 3.9%, 5.9%, and 8.0% of total encryption time, respectively. This proportion is markedly lower than at the 256 × 256 resolution, since ML-KEM’s cost is independent of image size, while the image-layer cost scales with pixel count; at 512 × 512, four times as many pixels are processed per channel, which dilutes the relative contribution of the fixed-cost key encapsulation step. This reflects the three independent per-channel encapsulations performed for an RGB image, each of which is more costly for higher-security ML-KEM parameter sets. While no longer negligible at the strongest security level, ML-KEM remains a minority contributor to total latency even for ML-KEM-1024, and the total encryption time across all nine (image; variant) configurations remains within a narrow 227–258 ms range for a 512×512×3 image, confirming that the choice of ML-KEM variant can be made primarily on security grounds without a disproportionate performance penalty.

It should be noted that the timing figures reported above reflect a pure-Python implementation (*kyber-py* for ML-KEM, NumPy for pixel-level operations), which is orders of magnitude slower than optimized C, assembly, or hardware (FPGA/ASIC) implementations of the same underlying algorithms, including production-grade ML-KEM libraries such as *liboqs*. Consequently, the absolute millisecond values reported here should not be directly compared against schemes in the literature that report timings from compiled or hardware-accelerated implementations (e.g., Table 1); such comparisons would systematically favor schemes implemented in lower-level languages regardless of their underlying computational complexity. The timing analysis in this study is intended to characterize the *relative* cost distribution within a single, consistent implementation, rather than to establish absolute performance claims relative to prior work. A production deployment would be expected to see substantially lower absolute latencies across all reported configurations.

### 5.3. Encryption Results

**Figure 7:**
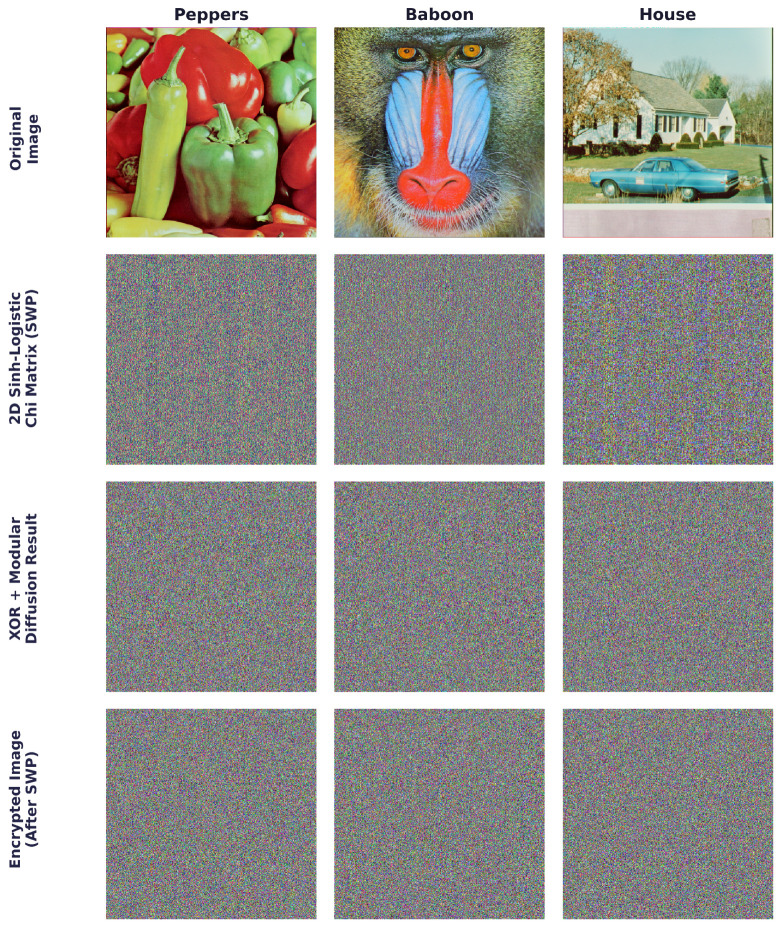
Step-by-step encryption visualization of proposed scheme.

Figure 7 illustrates the step-by-step encryption process for three RGB test images (Peppers, Baboon, and House). Row 1 shows the original images, Row 2 displays the keystream generated from the 2D Sinh-Logistic chaotic map, Row 3 presents the intermediate state after XOR and modular diffusion, and Row 4 shows the final encrypted images after applying SWP. All encrypted images in the final row are visually indistinguishable from random noise. The gradual transformation from structured plaintext to the final ciphertext via chaotic key flow and propagation visually confirms that the combined effect of the two encryption stages completely eliminates all spatial structure and statistical regularity.

**Figure 8:**
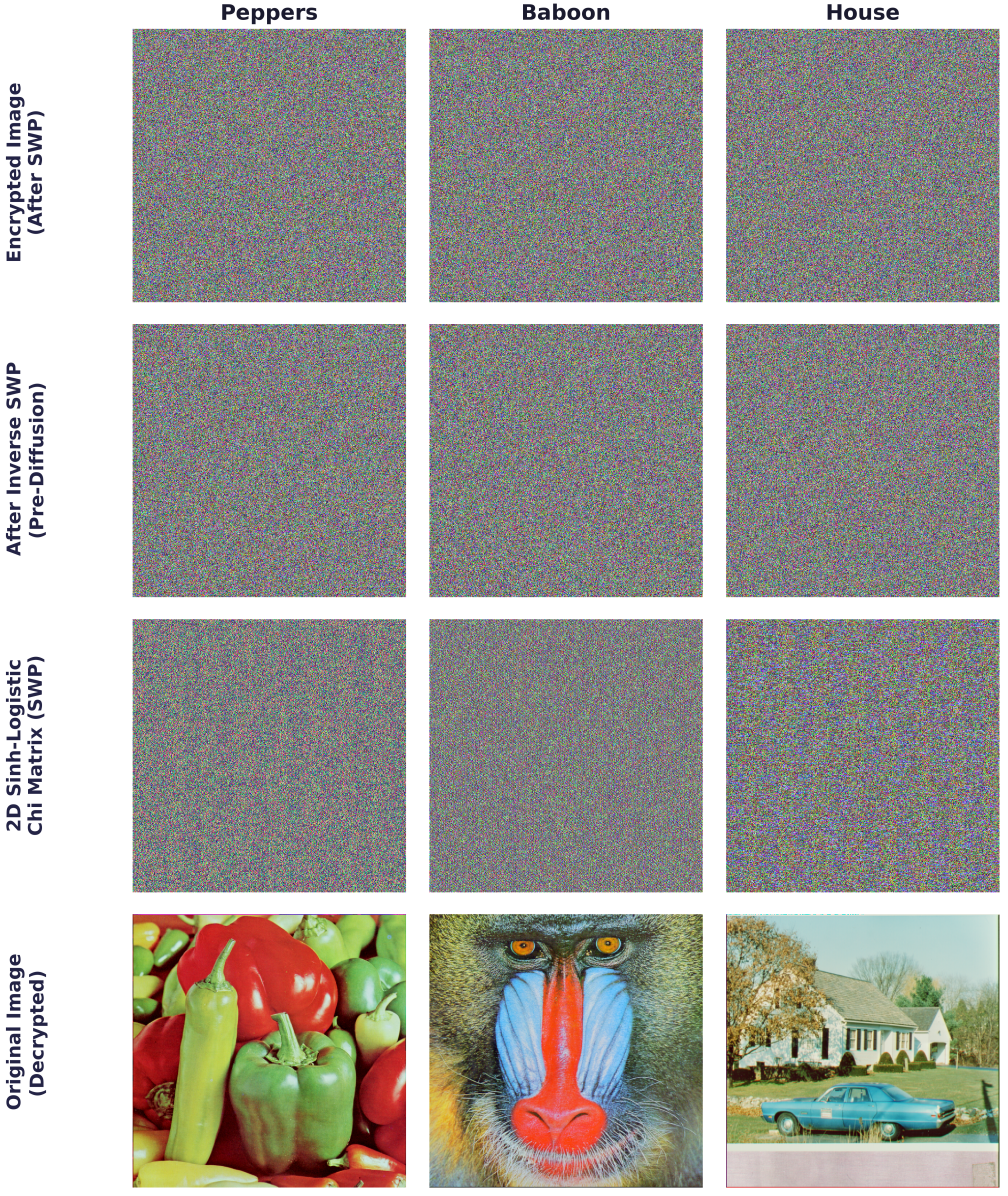
Step-by-step decryption visualization via inverse SWP, inverse modular step, and inverse XOR.

Figure 8 visualizes the step-by-step decryption process for all three RGB test images. Naturally, the decryption steps are the sequential reverse execution of the encryption steps. Decryption begins with the ciphertext image. First, a reverse SWP permutation is applied, restoring the spread pixel sequence to its original spatial order and keeping the propagation mask active. The third row shows the intermediate result after the inverse modular step, where the additional feedback term is removed but the XOR mask is still present. The last row shows the original image recovered after the inverse XOR step.

This lossless recovery is guaranteed thanks to the reversibility of both the SWP permutation and the feedback diffusion chain. Naturally, for the decryption steps to work correctly, the correct ML-KEM secret key sk must be available, the combined key must be regenerated, and all encryption parameters must be rederived.

### 5.4. Salt-And-Pepper and Gaussian Noise Attacks

**Figure 9:**
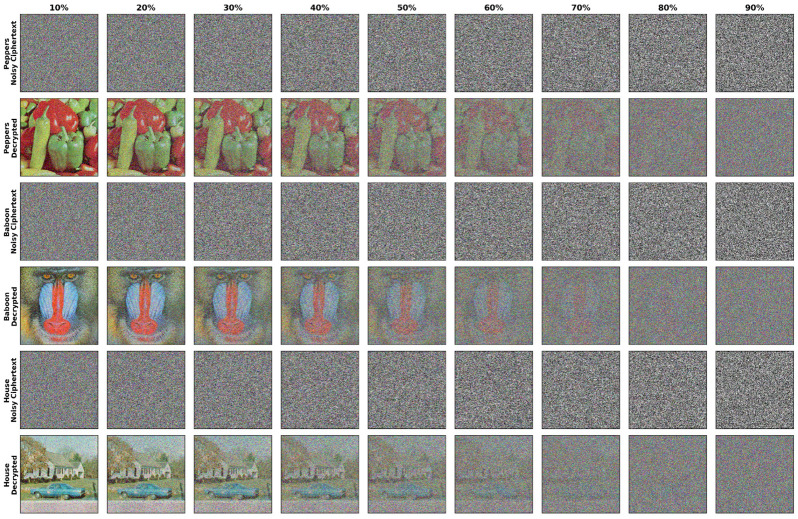
Salt-and-pepper noise attack results for Peppers, Baboon, and House (512 × 512), with the corrupted pixel fraction swept from 10% to 90%. For each image, the top row shows the noisy ciphertext, and the bottom row shows the corresponding decrypted image.

**Figure 10:**
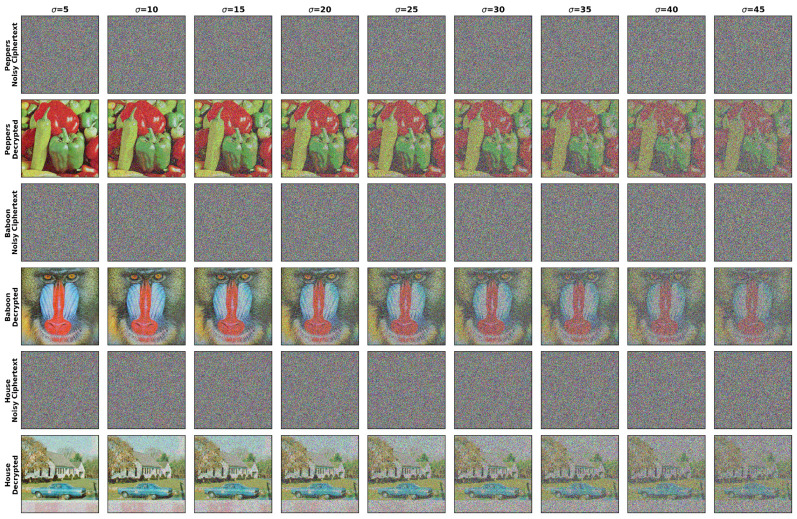
Gaussian noise attack results for Peppers, Baboon, and House (512 × 512), with the noise standard deviation swept from σ = 5 to σ = 45. For each image, the top row shows the noisy ciphertext, and the bottom row shows the corresponding decrypted image.

The results for both salt-and-pepper and Gaussian noise attacks are shown in Figure 9 and Figure 10.

In the proposed method, since the diffusion process advances pixel dependencies sequentially, salt-and-pepper noise leads to more localized and sharp distortions in the decrypted image. In contrast, the effect of Gaussian noise is more evenly distributed throughout the image. This difference is presented in Table 6 and Table 7 and Figure 9 and Figure 10.

This behavior reflects an inherent robustness–diffusion trade-off rather than a general weakness. The same sequential feedback chaining (Section 3.4) that produces the high avalanche effect and near-ideal NPCR values reported in Section 5.1 also causes a single corrupted pixel to propagate its effect to all subsequent pixels in the chain during decryption, since the inverse diffusion (Equations (14) and (15)) relies on the preceding ciphertext value. Consequently, both PSNR and structural similarity degrade substantially at higher noise levels: at 20% salt-and-pepper noise, PSNR drops to approximately 12.5 dB and SSIM falls below 0.10 (Table 6), and at Gaussian noise with σ=40, SSIM falls to approximately 0.04–0.12 across the three test images (Table 7). This degradation should be interpreted as a direct consequence of the diffusion design rather than as evidence of general resilience; the low SSIM values in particular indicate that structural content is not meaningfully preserved once ciphertext corruption exceeds a few percent. Applications requiring robustness to channel noise or transmission errors would need to combine the proposed scheme with an error-correction or retransmission layer, which lies outside the scope of the present design.

### 5.5. Data-Cut (Occlusion) Attack

**Figure 11:**
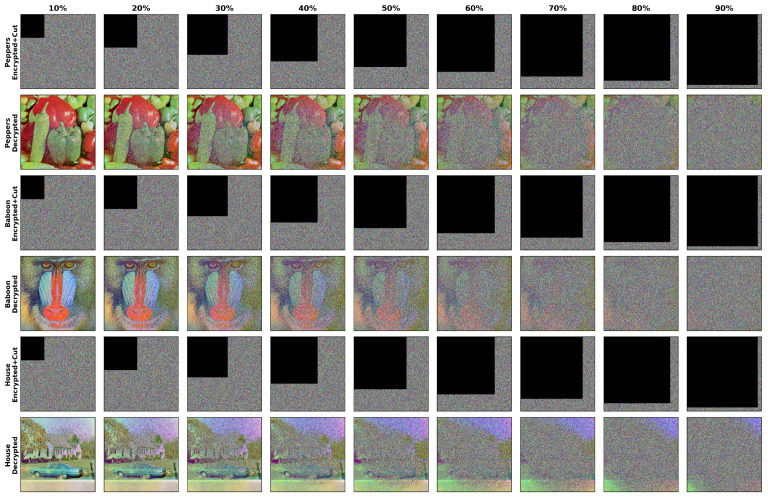
Data-cut (occlusion) attack results for Peppers, Baboon, and House (512 × 512), with the cut area swept from 10% to 90% of the total image. For each image, the top row shows the ciphertext with the cut region shown in black, and the bottom row shows the corresponding decrypted image.

To evaluate resistance against ciphertext data loss, a data-cut (occlusion) attack was performed following standard practice in the image-encryption literature: a square block in the top-left corner of each ciphertext was zeroed out (simulating lost or corrupted transmission data), with the cut area swept from 10% to 90% of the total image area in 10% increments, and the tampered ciphertext was decrypted using the correct key. Results are summarized in Table 8 and illustrated in Figure 11.

Unlike diffusion-only ciphers, in which a single corrupted ciphertext position propagates forward through the entire decryption chain, the proposed inverse-diffusion formulation (Equations (14) and (15)) depends only on $d_{i}$ and $d_{i-1}$ at each position; a single corrupted diffusion-array entry therefore corrupts exactly two adjacent plaintext positions rather than every subsequent one. However, because the cut region is contiguous in ciphertext space while the inverse SWP permutation maps ciphertext positions to scattered, non-contiguous positions in the diffusion array (Section 3.5), the resulting pixel errors are spread throughout the entire decrypted image rather than confined to the cut region, as shown in Figure 11. Image quality degrades gracefully with increasing cut area (PSNR from approximately 16 dB at 10% cut down to approximately 8 dB at 90% cut), and the decrypted images remain visually recognizable up to approximately 30–40% data loss across all three test images. This behavior is consistent with a substantial body of the chaos-based image-encryption literature, where graceful degradation under partial data loss is reported as a desirable property for transmission over lossy or adversarial channels [33]. At the same time, this behavior confirms that the scheme, as evaluated, does not provide cryptographic ciphertext integrity: no MAC or authenticated-encryption layer is included (Section 6), and an active adversary who tampers with the ciphertext is not cryptographically prevented from obtaining a partially reconstructed image. The distortion is nonetheless globally visible rather than silent or confined to the tampered region, providing a practical, if informal, tamper-evidence property even in the absence of formal integrity verification.

### 5.6. IND-CPA Security Analysis

#### 5.6.1. Formal Security Model

The proposed method has been evaluated within the scope of the IND-CPA (Indistinguishability under Chosen Plaintext Attack) security model used for image encryption in cryptanalysis studies. The aim of this analysis is to determine whether an attacker can distinguish which of two different equal-size plaintext RGB images is encrypted. If they can correctly guess which of the two images is encrypted with a higher probability than a random guess, this indicates a traceable lead in the proposed scheme, meaning there is a security vulnerability. The proposed nonce-based key derivation is designed to prevent this distinguishing attack: because the transmitted metadata contains only random, channel-specific nonces rather than any plaintext-derived value, an adversary cannot use the metadata to determine which image was encrypted.

The IND-CPA scenario is defined as follows:

**Setup**: A public key (pk) and a private key (sk) are generated using the ML-KEM.Keygen() algorithm. The public key is given to the attacker.

**Selection**: The attacker selects two different images ($m_{0}$ and $m_{1}$) with the same dimensions (M×N×3) and sends them to the system.

**Encryption**: The system selects a random bit b∈{0,1} and encrypts the selected image $m_{b}$ using the public key. The complete ciphertext package is transmitted to the adversary, consisting of the following:

- Channel-specific ML-KEM ciphertexts $ct_{\mathrm{kem},R}$, $ct_{\mathrm{kem},G}$, and $ct_{\mathrm{kem},B}$
- Encrypted image ($C_{b}$)
- Auxiliary metadata: channel-specific nonces $nonce_{R}$, $nonce_{G}$, and $nonce_{B}$

The adversary observes all transmitted components.

**Prediction**: The attacker guesses which image was encrypted and generates a value of $b^{\prime }$.

**Success Status**: If the attacker’s guess is correct ($b^{\prime }=b$), the attack is considered successful.

The attacker’s success is measured as follows: In the case of a random guess, the probability of guessing correctly is 1/2. The success difference above this value is defined as an “advantage”. A method is considered IND-CPA secure if this advantage remains negligible for all reasonable (polynomial-time) attackers.

#### 5.6.2. Security Argument

The IND-CPA security of the proposed scheme is based on three fundamental features:

1. Security of ML-KEM:
  The ML-KEM used in the key generation phase provides IND-CCA2 security. This security is based on the difficulty of the MLWE (Module Learning with Errors) problem. MLWE is a mathematical problem where it is difficult to extract hidden information from faulty (noise-added) linear equations. The difficulty of solving this problem ensures the security of the system. Furthermore, the fresh channel-specific shared secrets generated by independent ML-KEM encapsulations ensure that the encryption outputs are independent of each other, even if the same RGB image is encrypted repeatedly.
2. Plaintext-independent key derivation:
  The scheme generates each channel-specific encryption key as follows:
  $$\begin{array}{ccc} combined\text{\_}key_{ch}\; & = & \mathrm{HKDF}(\\ \; & \; & \left.ss_{ch},\;salt=nonce_{ch},\;info=“\mathrm{ND}\text{-}\mathrm{CPA}\text{-}\mathrm{Key}\text{-}”\|ch\right) \end{array}$$
  where $nonce_{ch}$ is an independent random 32-byte value generated for each color channel. Unlike traditional plaintext-dependent approaches, this construction ensures that the channel-specific combined keys reveal no information about the encrypted image. The channel-specific nonces are transmitted with the ciphertext package but do not compromise security, since they are independent of the plaintext content. This plaintext independence is the critical requirement for IND-CPA security.
3. Deterministic but unpredictable parameter derivation:
  All channel-specific encryption parameters $\left(x_{0,ch},y_{0,ch},\alpha_{ch},\beta_{ch}\right)$, keystreams $\left(key1_{ch},key2_{ch}\right)$, and SWP parameters $\left(c_{x,ch},c_{y,ch},\lambda_{ch}\right)$ are derived deterministically from $combined\text{\_}key_{ch}$ using HKDF with distinct context strings. While deterministic given $combined\text{\_}key_{ch}$, these parameters appear statistically independent and uniformly random to an attacker without key knowledge. This prevents correlation-based attacks on the encryption parameters.

These three features together support plaintext independence, enabling near-random adversarial success in the IND-CPA-style indistinguishability experiment.

#### 5.6.3. Experimental IND-CPA Game Simulation

To experimentally validate the IND-CPA security of the proposed scheme, we performed IND-CPA game simulations over 5000 rounds per image using four independent distinguishers (histogram-distance, chi-square uniformity, correlation-based, and a learned classifier trained via chosen-plaintext oracle access), across three standard RGB test images (Peppers, Baboon, and House) instantiated with ML-KEM-512.

**Figure 12:**
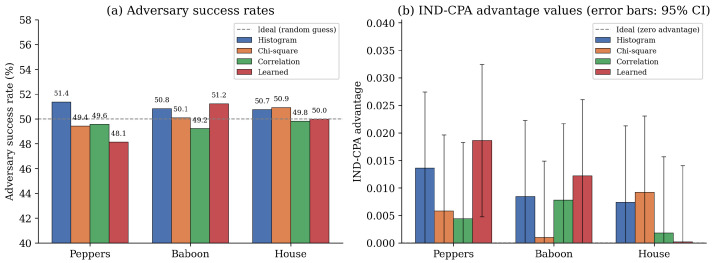
IND-CPA game simulation results over 5000 rounds per image using four independent distinguishers (histogram, chi-square, correlation, and a learned classifier); ML-KEM-512. (**a**) shows the adversary success rate (%) for each image–distinguisher pair relative to the ideal random-guess baseline of 50%; (**b**) presents the corresponding IND-CPA advantage values. Error bars in (**b**) denote 95% Wilson confidence intervals; all intervals are wide relative to the point estimates, indicating the observed advantages are statistically indistinguishable from zero.

Results: The adversary success rate was 48.14% (Peppers, worst case) (advantage: 0.0186, the maximum observed across all three images and four distinguishers), which is statistically equivalent to random guessing (50%) and confirms IND-CPA security. Comparative results are presented in Table 9 and visualized in Figure 12.

### 5.7. Key Space Analysis

The resistance to brute-force attacks depends on the size of the key space. The session key is derived from the 32-byte ML-KEM-512 shared secret using HKDF-SHA256 with a random nonce. This results in an effective key space of $2^{256}$, matching AES-256 and significantly exceeding the $2^{128}$ level considered sufficient for classical security.

However, the size of the key space alone is not a sufficient indicator of security. In the context of quantum computing, Shor’s algorithm threatens classical asymmetric systems, while Grover’s algorithm reduces symmetric key security to the square root, effectively halving the bit security level (e.g., $2^{256}$ key space provides $2^{128}$ quantum security). The proposed scheme’s ML-KEM-512 variant provides $2^{128}$ quantum security, exceeding NIST Category 1 requirements. Furthermore, the scheme’s empirically validated IND-CPA security demonstrates that a large key space must be coupled with semantic security. ML-KEM-768 and ML-KEM-1024 variants offer $2^{192}$ and $2^{256}$ quantum security, respectively.

Table 10 compares the key space of the proposed method with similar studies in the literature. Most existing chaos-based methods claim key spaces between $2^{128}$ and $2^{485}$ but rely on classical key mechanisms vulnerable to quantum attacks. The proposed method is the only one using NIST-standardized post-quantum KEM (ML-KEM/FIPS 203), providing quantum-resistant key exchange with $2^{256}$ classical security and $2^{128}$ quantum security.

Overall, it was observed that the encrypted images could not be distinguished from random noise using the tested distinguisher family, evaluated with ML-KEM-512. The results obtained empirically support the IND-CPA security of the proposed method.

### 5.8. Key Sensitive Analysis

In a secure encryption scheme, even a very small change in the key should produce a completely different encrypted output. To test this, the same RGB test image is encrypted with two keys differing by only one bit (the least significant bit of the last byte is changed), and the two encrypted results are compared using quantitative metrics as presented in Table 11.

The two resulting encrypted images ($C_{1}$ and $C_{2}$), although produced from keys differing by a single bit, yield pixel values that are almost completely different. Quantitative analysis across all RGB test images (Peppers, Baboon, and House) confirms this high key sensitivity. Specifically, the NPCR values exceed 99.61% in all test images, surpassing the ideal threshold of 99.6094%. The UACI values are close to the theoretical ideal of 33.4635%, with an average value of 33.4607%. Furthermore, the near-zero correlation between $C_{1}$ and $C_{2}$ indicates that the two outputs are statistically independent.

This high sensitivity stems from the key derivation process and the encryption scheme structure. A single-bit change in the key is propagated throughout the HKDF derivation, completely altering the chaotic initial values, keystreams, and permutation indices. As a result, the encryption process proceeds along an entirely different trajectory, and the resulting output becomes statistically independent from the previous encrypted image.

## 6. Discussion

Measures such as NPCR, UACI, and Shannon entropy are often presented as evidence that a scheme is safe; however, these measures are statistical tests and are not sufficient on their own for safety. NPCR quantifies how many pixel values change when a single plaintext pixel is altered—a useful measure of diffusion, but not a guarantee against a computationally bounded adversary. More critically, it has been observed that near-ideal NPCR and UACI values can be produced by transformations that offer little actual security; a sufficiently randomized but cryptographically weak permutation can yield NPCR > 99.6% while still being vulnerable to chosen-plaintext attacks. Entropy, similarly, measures the uniformity of pixel value distribution in the ciphertext but says nothing about whether an adversary can distinguish that ciphertext from one produced under a different key. In other words, a scheme can pass every standard statistical test and still fail a basic IND-CPA experiment. This gap between statistical indistinguishability and cryptographic indistinguishability is rarely acknowledged in the chaos-based image-encryption literature and is almost never empirically tested.

The proposed scheme addresses this gap through its nonce-based key derivation, which structurally prevents metadata-based plaintext leakage. By conducting an IND-CPA simulation over 5000 rounds per test image, using four independent distinguishers (histogram-distance, chi-square uniformity, pixel correlation, and a learned classifier trained via chosen-plaintext oracle access), we provide empirical measurements of adversarial advantage for a chaos-based image cipher grounded in a post-quantum KEM. The scheme achieves a maximum adversary advantage of 0.0186 across all tested images and distinguishers, statistically consistent with random guessing, suggesting, within the tested adversarial setting, that the ciphertext carries no exploitable information about the plaintext under a chosen-plaintext attack model. This result is not achievable by reporting NPCR or entropy alone; it requires constructing explicit adversaries, running a security experiment, and measuring the advantage directly.

With that context established, the standard metrics do serve a useful role as sanity checks. The proposed scheme achieved entropy above 7.996, NPCR above 99.59%, and UACI close to 33.4635% across all three test images (Peppers, Baboon, and House)—results that are competitive with the best-performing schemes in the literature.

Experimental results demonstrate that the proposed scheme achieves robust performance in both classical statistical metrics and the IND-CPA security criterion. A fundamental challenge in chaos-based cryptography is dynamic degradation under finite-precision arithmetic. While continuous-domain chaotic maps exhibit ideal properties, digital implementations using IEEE 754 double-precision (64-bit) representation operate with limited numerical accuracy. The proposed system requires 786,432 iterations for 512×512 RGB image encryption, which remains below the $10^{6}$–$10^{7}$ iteration threshold where precision-induced corruption typically emerges [11]. More importantly, the empirical results presented above (entropy >7.996, correlation <0.009, NPCR ≥99.59%, and IND-CPA security with a maximum adversary advantage of 0.0186) confirm that finite precision does not introduce exploitable weaknesses in practice. The cryptographic strength derives from the combined architecture (chaotic mixing, feedback diffusion, and SWP permutation), providing robustness even if individual components experience minor precision effects. The IND-CPA validation provides the strongest confirmation: no distinguishability issue was observed in the conducted empirical tests.

The integration of ML-KEM addresses a second structural weakness in the current literature: the continued reliance on classical key exchange. ECDH, which appears in several recent chaos-based encryption works, is efficiently broken by Shor’s algorithm on a sufficiently powerful quantum computer. By grounding the key derivation in ML-KEM standardized as FIPS 203 by NIST in August 2024, the proposed scheme is quantum-resistant by construction. Importantly, ML-KEM provides IND-CCA2 security per the FIPS 203 specification, which implies IND-CPA for the key encapsulation layer. This means the scheme’s semantic security rests on two independent foundations: the empirical IND-CPA simulation at the cipher level and the formal IND-CCA2 guarantee at the key exchange level.

The timing analysis across RGB test images (Peppers, Baboon, and House) shows that ML-KEM’s contribution scales with the chosen security level, ranging from approximately 3.9% (ML-KEM-512) to 8.0% (ML-KEM-1024) of total encryption latency, while the remaining majority is dominated by the chaotic-map iteration and feedback diffusion phases (Section 5.2). This confirms that the post-quantum upgrade remains a minority cost and that all three ML-KEM variants keep the total encryption time within a narrow 227–258 ms range. Therefore, the proposed scheme achieves post-quantum key establishment with a modest and predictable computational overhead compared to classical chaos-based methods, making variant selection primarily a security-driven decision rather than a performance trade-off. Future optimization efforts should focus on parallelizing the chaotic-map computation and diffusion operations, which together dominate the image-processing layer.

The system independently encrypts each color channel in RGB images, ensuring strong statistical security across all channels. Future work will develop a formal security proof reducing the system’s security to the hardness of the Module-LWE problem, complementing experimental results with provable security guarantees. Additionally, hardware performance evaluation will be conducted on FPGA and embedded ARM platforms for real-time deployment.

The diffusion stage (Equations (12) and (13)) relies solely on XOR and modular addition with serial feedback, without a nonlinear substitution (S-box) layer; avalanche propagation is therefore driven entirely by the chaining term $d_{i-1}$. In principle, this raises the question of whether an adversary with knowledge of a suffix of the plaintext could recover key material by working backward through the chain. In the proposed construction, however, each position *i* uses independent, HKDF-derived key bytes $key1_{i}$ and $key2_{i}$ that are never reused across positions or encryption instances; recovering $d_{i-1}$ and $p_{i}$ at a single position yields one equation in two unknowns ($key1_{i}$; $key2_{i}$), which is information-theoretically insufficient to recover either byte, let alone the underlying $combined\text{\_}key_{ch}$ from which they are derived. A more consequential limitation is that the scheme, as evaluated, provides confidentiality (IND-CPA) but not ciphertext integrity: no MAC or authenticated-encryption layer is included, so an active adversary capable of modifying the transmitted ciphertext could exploit the chaining structure to perform targeted bit-flipping or truncation attacks that remain undetected at decryption time. This places the scheme’s security guarantees within an IND-CPA (passive adversary) threat model, consistent with the analysis in Section 5.6, rather than an IND-CCA2 (active adversary) one. Incorporating a lightweight substitution layer and an authenticated-encryption construction (e.g., an HMAC- or AEAD-based integrity tag over the transmitted ciphertext and metadata) is a natural direction for future work and would be necessary before the scheme could be considered for deployment against active network adversaries.

## 7. Conclusions

This study presented a nonce-based post-quantum image-encryption framework integrating NIST-standardized ML-KEM post-quantum key encapsulation, 2D Sinh-Logistic chaotic-map-based key generation, and a novel permutation algorithm, SWP. The proposed system addresses a critical gap in the literature by providing strong statistical properties at the image layer while establishing a future-proof architecture through standardized post-quantum key management.

Extensive experimental results demonstrate that the proposed system exhibits strong performance across all key security metrics. Entropy values exceed 7.999, NPCR exceeds 99.59%, UACI ranges between 33.41% and 33.53%, close to the theoretical ideal, and pixel correlations are close to zero. These results are competitive with existing methods. Furthermore, in the IND-CPA simulation (5000 rounds per image, four independent distinguishers), the attacker advantage was observed to be a maximum of 0.0186. This indicates that the encrypted data does not leak any meaningful information about the plaintext. This study is, to our knowledge, one of the first to experimentally evaluate a NIST-standardized post-quantum KEM (FIPS 203) with a chaos-based encryption method under the IND-CPA framework.

Computational analyses on RGB test images (Peppers, Baboon, and House) show that the contribution of the ML-KEM operation to the total encryption time scales with the security level, ranging from approximately 3.9% (ML-KEM-512) to 8.0% (ML-KEM-1024), with the remaining majority dominated by the chaotic-map iteration and feedback diffusion phases.Therefore, the proposed scheme achieves post-quantum security with a modest, predictable computational overhead compared to classical chaos-based methods. The fact that the ML-KEM-512, 768, and 1024 variants keep the total encryption time within a narrow range (227–258 ms) in practice makes it possible to adapt the system to different security needs (NIST Category 1, 3, or 5) without a disproportionate performance penalty.

RGB color image support has been successfully implemented through per-channel independent encryption, maintaining statistical security across all channels. Future studies may provide a Module-LWE-based formal reduction proof to complement the empirical IND-CPA results and extend the methodology to real-time video stream encryption requiring frame dependency analysis and streaming protocol integration for resource-constrained or latency-critical applications. The integration of post-quantum key exchange into chaos-based encryption methods is a critical development in this field, and this work demonstrates its practical feasibility through ML-KEM (FIPS 203) integration.

## Figures and Tables

**Table 2:** Security metrics, definitions, and their ideal values for image-encryption evaluation.

Metric	Definition	Ideal Value
Shannon Entropy	$H=-\sum p\left(i\right)\log_{2}p\left(i\right)$, ideal value 8.0 bit/px for uniformly distributed ciphertext.	8.0 bit/px
NPCR	Number of Pixels Change Rate: percentage of pixels that differ between C(m) and $C(m^{\prime })$, where $m^{\prime }$ differs from *m* by exactly one pixel.	≈99.6094%
UACI	Unified Average Changing Intensity: normalized mean absolute difference between C(m) and $C(m^{\prime })$.	≈33.4635%
Correlation Coefficient	Adjacent pixel correlation in horizontal, vertical, and diagonal directions.	≈0
IND-CPA Advantage	Adversary’s advantage in the IND-CPA game: $Adv=|\mathrm{Pr}[b^{\prime }=b]-0.5|$.	≈0
Key Space	≥2^128^ (brute-force resistant).	≥2^128^
Key Sensitivity	A single-bit change in the key produces a completely different ciphertext, verified by NPCR and UACI metrics above.	High

**Table 3:** Performance analysis of the proposed encryption scheme using RGB test images (Peppers, Baboon, and House, 512 × 512) and comparison with ideal values.

Image	Channel	$H_{\mathrm{cipher}}$	NPCR (%)	UACI (%)	Corr. H	Corr. V	Corr. D
	R	7.9993	99.62	33.47	−0.0002	0.0001	−0.0008
Peppers	G	7.9993	99.60	33.45	0.0011	0.0007	−0.0027
	B	7.9994	99.62	33.48	−0.0006	0.0023	0.0019
	R	7.9993	99.61	33.53	0.0037	−0.0022	0.0017
Baboon	G	7.9992	99.59	33.44	−0.0034	0.0018	0.0027
	B	7.9994	99.61	33.43	−0.0002	0.0004	−0.0001
	R	7.9993	99.62	33.41	0.0011	−0.0038	0.0046
House	G	7.9994	99.61	33.49	0.0009	0.0014	0.0002
	B	7.9993	99.61	33.45	−0.0019	0.0010	−0.0007
Ideal	—	8.0000	≥99.61	≈33.46	≈0.00	≈0.00	≈0.00

**Table 4:** Correlation coefficient results of the proposed scheme across 14 standard SIPI test images.

Image	Hor. Corr.		Ver. Corr.		Dia. Corr.	
	Plaintext	Cipher	Plaintext	Cipher	Plaintext	Cipher
4.1.01	0.9740	−0.0025	0.9657	−0.0001	0.9515	−0.0004
4.1.02	0.9395	−0.0013	0.9567	−0.0011	0.9089	0.0089
4.1.03	0.9761	0.0024	0.9142	0.0060	0.8973	0.0020
4.1.04	0.9700	0.0015	0.9848	−0.0007	0.9572	−0.0026
4.1.05	0.9782	−0.0050	0.9529	0.0059	0.9360	0.0025
4.1.06	0.9682	0.0010	0.9451	0.0049	0.9300	0.0027
4.1.07	0.9788	−0.0007	0.9824	0.0007	0.9647	0.0032
4.1.08	0.9728	0.0052	0.9757	0.0007	0.9495	0.0039
4.2.01	0.9839	0.0019	0.9913	0.0023	0.9773	0.0009
4.2.03	0.8665	0.0000	0.7587	0.0003	0.7262	-0.0042
4.2.05	0.9663	−0.0007	0.9641	−0.0016	0.9370	-0.0011
4.2.06	0.9751	−0.0002	0.9715	0.0030	0.9578	−0.0023
4.2.07	0.9768	−0.0029	0.9792	0.0003	0.9639	0.0002
house	0.9485	0.0018	0.9578	−0.0025	0.9135	0.0031
Average	0.9625	0.0000	0.9500	0.0013	0.9265	0.0012

**Table 5:** Image-wise ML-KEM variant security-performance trade-off (Peppers, Baboon, and House, 512 × 512).

Image	Variant	KEM (ms)	Image Layer (ms)	Total Enc. (ms)	Dec. (ms)
Peppers	ML-KEM-512	8.82	217.74	226.57	227.37
Peppers	ML-KEM-768	13.76	221.46	235.21	231.47
Peppers	ML-KEM-1024	20.21	233.65	253.86	250.36
Baboon	ML-KEM-512	9.29	223.07	232.36	221.69
Baboon	ML-KEM-768	15.00	238.55	253.55	247.33
Baboon	ML-KEM-1024	20.43	237.37	257.80	253.54
House	ML-KEM-512	9.35	228.74	238.08	227.08
House	ML-KEM-768	14.80	240.27	255.07	249.70
House	ML-KEM-1024	20.19	231.97	252.16	249.80

**Table 6:** Salt-and-pepper noise attack: PSNR (dB) and SSIM of decrypted images versus fraction of ciphertext pixels corrupted, for Peppers, Baboon, and House (512 × 512).

Image	Metric	10%	20%	30%	40%	50%	60%	70%	80%	90%
Peppers	PSNR (dB)	15.3	12.5	11.0	10.0	9.3	8.8	8.5	8.3	8.1
	SSIM	0.193	0.100	0.064	0.044	0.030	0.021	0.016	0.011	0.009
Baboon	PSNR (dB)	16.0	13.2	11.7	10.7	10.0	9.5	9.2	9.0	8.8
	SSIM	0.419	0.251	0.164	0.110	0.073	0.047	0.030	0.018	0.011
House	PSNR (dB)	15.7	12.9	11.4	10.4	9.7	9.2	8.9	8.7	8.5
	SSIM	0.272	0.153	0.099	0.067	0.045	0.030	0.021	0.015	0.010

**Table 7:** Gaussian noise attack: PSNR (dB) and SSIM of decrypted images versus noise standard deviation σ, for Peppers, Baboon, and House (512 × 512).

Image	Metric	σ = 5	σ = 10	σ = 15	σ = 20	σ = 25	σ = 30	σ = 35	σ = 40	σ = 45
Peppers	PSNR (dB)	17.3	14.6	13.0	11.9	11.1	10.4	10.0	9.6	9.3
	SSIM	0.253	0.156	0.115	0.090	0.072	0.059	0.049	0.040	0.034
Baboon	PSNR (dB)	18.4	15.6	14.0	12.9	12.1	11.5	11.0	10.5	10.2
	SSIM	0.537	0.391	0.310	0.254	0.211	0.175	0.144	0.118	0.098
House	PSNR (dB)	18.1	15.3	13.7	12.5	11.7	11.0	10.5	10.1	9.8
	SSIM	0.354	0.240	0.184	0.148	0.121	0.100	0.083	0.068	0.056

**Table 8:** Data-cut attack: PSNR (dB) and SSIM of decrypted images versus percentage of ciphertext area cut, for Peppers, Baboon, and House (512 × 512).

Image	Metric	10%	20%	30%	40%	50%	60%	70%	80%	90%
Peppers	PSNR (dB)	15.9	13.1	11.5	10.4	9.6	9.1	8.7	8.4	8.2
	SSIM	0.508	0.334	0.227	0.157	0.103	0.063	0.038	0.021	0.011
Baboon	PSNR (dB)	16.3	13.5	12.0	11.0	10.3	9.7	9.3	9.1	8.9
	SSIM	0.586	0.408	0.294	0.209	0.142	0.086	0.051	0.028	0.015
House	PSNR (dB)	16.0	13.3	11.9	10.9	10.2	9.6	9.2	8.9	8.6
	SSIM	0.585	0.424	0.310	0.218	0.146	0.093	0.060	0.035	0.016

**Table 9:** IND-CPA game results across four independent distinguishers (5000 rounds each; ML-KEM-512).

Image	Histogram	Chi-Square	Correlation	Learned	Max Adv.
Peppers	0.0136	0.0058	0.0044	0.0186	0.0186
Baboon	0.0084	0.0010	0.0078	0.0122	0.0122
House	0.0074	0.0092	0.0018	0.0002	0.0092

**Table 10:** Key space comparison with related image-encryption schemes.

Reference	Key Space	Type	PQC	Chaotic Map
Alghamdi et al. [32]	$2^{256}$	Lightweight	×	Logistic Map
İnce et al. [33]	$2^{384}$	Lightweight	×	Logistic + Tent
Ghorbani et al. [61]	$2^{256}\times R$	Standard	×	LSTC Map
Shah et al. [62]	$2^{192}$	Lightweight	×	Logistic Map
Deb & Bhuyan [63]	$2^{256}$	Medium	×	Logistic + Tent
Kumari & Mondal [64]	$2^{256}$	Medium	×	NLFSR
Zhang et al. [65]	$2^{485}$	Medium	×	Chen’s System
Proposed	$2^{256}$	Standard	√	2D Sinh-Logistic

**Table 11:** Comparative analysis of image-encryption schemes: statistical metrics and security model properties.

Metric	[54]	[32]	[33]	[13]	Proposed
Year	2021	2022	2025	2025	2026
Key Exchange	Chebyshev	None	None	ECDH (classical)	ML-KEM (FIPS 203)
Entropy	N/R	7.9992	7.9993	≈7.999	7.9972
NPCR (%)	N/R	99.6153	99.6122	>99.61	99.6140
UACI (%)	N/R	33.4718	33.4690	>33.46	33.4731
Key Space	N/R	$2^{256}$	$2^{384}$	$10^{256}$	$2^{256}$
IND-CPA	✓	×	×	×	✓
PQC	×	×	×	×	✓

## Data Availability

The original contributions presented in this study are included in this article. Further inquiries can be directed to the corresponding author.

## Abbreviations

The following abbreviations are used in this manuscript:

PQC	Post-Quantum Cryptography
ML-KEM	Module-Lattice-Based Key-Encapsulation Mechanism
FIPS	Federal Information Processing Standards
NIST	National Institute of Standards and Technology
MLWE	Module Learning With Errors
HKDF	HMAC-based Key Derivation Function
HMAC	Hash-based Message Authentication Code
SHA	Secure Hash Algorithm
SWP	Seismic Wave Permutation
IND-CPA	Indistinguishability under Chosen Plaintext Attack
IND-CCA2	Indistinguishability under Adaptive Chosen Ciphertext Attack
NPCR	Number of Pixels Change Rate
UACI	Unified Average Changing Intensity
RGB	Red, Green, Blue
KEM	Key Encapsulation Mechanism
ECDH	Elliptic Curve Diffie–Hellman
PRK	Pseudo-Random Key
OKM	Output Keying Material
FPGA	Field-Programmable Gate Array
ARM	Advanced RISC Machine

## References

[1] Ye C., Shi L., Tan S., Wang J., Zuo Q., Feng W. (2026). A controllable medical image security scheme using selective encryption and watermarking for bit-planes in the TSH domain. Cybersecurity.

[2] Alsahafi Y.S., Sarhan A.Y., Elnabawy Y.M., Hosny K.M. (2025). A new algorithm for multiple medical image encryption based on stacked representation and block division. Sci. Rep..

[3] Belazi A., Mabrouk A.B. (2025). A refined sine-derived chaotic map for securing medical image encryption in telemedicine. Comput. Biol. Med..

[4] Inam S., Kanwal S., Amin E., Cheikhrouhou O., Hamdi M. (2025). Securing face images in UAV networks using chaos and DNA cryptography approach. Sci. Rep..

[5] Elias E.P., Santhanavijayan A. (2025). Secure satellite image transmission with dynamic encipherment and AuthKeX protocol. Adv. Space Res..

[6] Tbahriti S.-E., Saad A., Boughanmi N. (2025). Advanced framework for securing satellite imagery in computationally constrained environments. Adv. Space Res..

[7] Li M., Zhu Y., Du R., Jia C. (2025). Verifiable encrypted image retrieval with reversible data hiding in cloud environment. IEEE Trans. Cloud Comput..

[8] Zhang X., Wang H., Lu J., Li T., Wang C. (2026). A Bloom filter-based dynamic symmetric searchable encryption scheme over cloud data. Cybersecurity.

[9] Ren L., Zhang D. (2025). Integrating visual cryptography for efficient and secure image sharing on social networks. Appl. Sci..

[10] Ye C., Tan S., Wang J., Shi L., Zuo Q., Feng W. (2025). Social image security with encryption and watermarking in hybrid domains. Entropy.

[11] Zhang B., Liu L. (2023). Chaos-based image encryption: Review, application, and challenges. Mathematics.

[12] Zheng Z. (2022). Modern Cryptography Volume 1: A Classical Introduction to Informational and Mathematical Principle.

[13] Zhiqiang H., Rauf A., Nazir A., Tchier F., Aslam A. (2025). Design and analysis of a secure image encryption algorithm using proposed non-linear RN chaotic system and ECC/HKDF. Sci. Rep..

[14] İnce K. (2024). Exploring the potential of deep learning and machine learning techniques for randomness analysis to enhance security on IoT. Int. J. Inf. Secur..

[15] Kaya M.S., İnce K. (2024). Benchmarking various 1D chaotic maps for lightweight pseudo-random number generation. Proceedings of the 2024 8th International Artificial Intelligence and Data Processing Symposium (IDAP), Malatya, Turkey, 21–22 September 2024.

[16] Shor P.W. (1994). Algorithms for quantum computation: Discrete logarithms and factoring. Proceedings of the 35th Annual Symposium on Foundations of Computer Science, Santa Fe, NM, USA, 20–22 November 1994.

[17] Grover L.K. (1996). A fast quantum mechanical algorithm for database search. Proceedings of the 28th Annual ACM Symposium on Theory of Computing, Philadelphia, PA, USA, 22–24 May 1996.

[18] (2024). Module-Lattice-Based Key-Encapsulation Mechanism Standard.

[19] Bos J., Ducas L., Kiltz E., Lepoint T., Lyubashevsky V., Schanck J.M., Schwabe P., Seiler G., Stehlé D. (2018). CRYSTALS-Kyber: A CCA-secure module-lattice-based KEM. Proceedings of the 2018 IEEE European Symposium on Security and Privacy, London, UK, 24–26 April 2018.

[20] Cheng S., Chen J., Li J., Yao K., Gao S., Rui K., Cui Y. (2025). Optimized design and implementation of CRYSTALS-KYBER based on MLWE. Secur. Commun. Netw..

[21] Kamatchi A., Arulprakasam R. (2026). Non-uniform second-order reversible cellular automata with chaos-based permutation for lossless image encryption. Nonlinear Dyn..

[22] Liu W., Sun K., Zhu C. (2016). A fast image encryption algorithm based on chaotic map. Opt. Lasers Eng..

[23] Sajjad M., Shah T., Haq T.U., Almutairi B., Xin Q. (2024). SPN based RGB image encryption over Gaussian integers. Heliyon.

[24] Tiwari A., Diwan P., Diwan T.D., Miroslav M., Samal S.P. (2025). A compressed image encryption algorithm leveraging optimized 3D chaotic maps for secure image communication. Sci. Rep..

[25] Truong D.L., Dang X.V., Dang T.N. (2023). Survivable free space optical mesh network using high-altitude platforms. Opt. Switch. Netw..

[26] Zhang P., Jie J., Liu Z., Dong K. (2025). Chaotic image encryption system as a proactive scheme for image transmission in FSO high-altitude platform. Photonics.

[27] Ulutas H. (2025). A novel memristor-based hyperchaotic hybrid encryption system with DNA for image encryption on the Jetson TX2. Sci. Rep..

[28] Şimşek C., Erkan U., Toktas A., Lai Q., Gao S. (2025). Hexadecimal permutation and 2D cumulative diffusion image encryption using hyperchaotic sinusoidal exponential memristive system. Nonlinear Dyn..

[29] Alexan W., Megalli Y. (2025). A new fast high dimensional and memristive hyperchaotic multiple image encryption method and its FPGA implementation. Discov. Electron..

[30] Alexan W., Hosny K., Gabr M. (2025). A new fast multiple color image encryption algorithm. Clust. Comput..

[31] Alexan W., Youssef M., Hussein H.H., Ahmed K.K., Hosny K.M., Fathy A., Mansour M.B.M. (2025). A new multiple image encryption algorithm using hyperchaotic systems, SVD, and modified RC5. Sci. Rep..

[32] Alghamdi Y., Munir A., Ahmad J. (2022). A lightweight image encryption algorithm based on chaotic map and random substitution. Entropy.

[33] İnce K., İnce C., Hanbay D. (2025). Random strip peeling: A novel lightweight image encryption framework for IoT devices based on colour planes stripe permutation. CAAI Trans. Intell. Technol..

[34] Zhou S., Wei Y., Wang S., Iu H.H.-C., Zhang Y. (2024). Novel chaotic image cryptosystem based on dynamic RNA and DNA computing. J. Appl. Phys..

[35] Artuğer F. (2024). A method for designing substitution boxes based on chaos with high nonlinearity. Wirel. Pers. Commun..

[36] Abdelaal M.A., Moustafa A.I., Kasban H., Saleh H., Abdallah H.A., Afifi M.Y.I. (2025). DNA-inspired lightweight cryptographic algorithm for secure and efficient image encryption. Sensors.

[37] Zhou M., Li X., Du W., Li J., Wei Z. (2025). Pixel-level and DNA-level image encryption method based on five-dimensional hyperchaotic system (PD5H). Entropy.

[38] Ouyang X., Tang S., Zeng Q., Fu Q., Qin S., Liu J., Luo Y. (2026). Image cryptosystem based on hyper-chaos and optimized typhoon wind field model. J. King Saud Univ. Comput. Inf. Sci..

[39] Kopp M.I., Samuilik I. (2025). Hidden attractors in a new 4D memristor-based hyperchaotic system: Dynamical analysis, circuit design, synchronization, and its applications. Mathematics.

[40] Udrescu M., Prodan L., Vladutiu M. (2006). Implementing quantum genetic algorithms: A solution based on Grover algorithm. Proceedings of the 3rd Conference on Computing Frontiers, Ischia, Italy, 3–5 May 2006.

[41] Khan Q., Purification S., Chang S.-Y. (2025). Post-quantum key exchange and subscriber identity encryption in 5G using ML-KEM (Kyber). Information.

[42] Olushola A., Meenakshi S.P. (2026). Design and implementation of an authenticated post-quantum session protocol using ML-KEM (Kyber), ML-DSA (Dilithium), and AES-256-GCM. Front. Phys..

[43] Bellare M., Canetti R., Krawczyk H. (1998). A modular approach to the design and analysis of authentication and key exchange protocols. Proceedings of the 30th Annual ACM Symposium on Theory of Computing (STOC ’98), Dallas, TX, USA, 24–26 May 1998.

[44] Khan M., Aljuaydi F., Said L., Amin M. (2025). A secure chaotic cryptosystem for thermal imaging: Logistic map-based encryption with substitution-diffusion and spatial decorrelation. J. Radiat. Res. Appl. Sci..

[45] Wang X., Chen X. (2021). An image encryption algorithm based on dynamic row scrambling and Zigzag transformation. Chaos Solitons Fractals.

[46] Wang X., Zhang J., Cao G. (2019). An image encryption algorithm based on ZigZag transform and LL compound chaotic system. Opt. Laser Technol..

[47] Jiang M., Yang H. (2023). Image encryption using a new hybrid chaotic map and spiral transformation. Entropy.

[48] İnce C., İnce K., Hanbay D. (2024). Novel image pixel scrambling technique for efficient color image encryption in resource-constrained IoT devices. Multimed. Tools Appl..

[49] Kaya M.S., İnce K. (2026). Knit scrambling: A novel image scrambling framework and its demonstration in image encryption. J. Inf. Secur. Appl..

[50] Zia U., McCartney M., Scotney B., Martinez J., AbuTair M., Memon J., Sajjad A. (2022). Survey on image encryption techniques using chaotic maps in spatial, transform and spatiotemporal domains. Int. J. Inf. Secur..

[51] Jiang Q., Yu S., Wang Q. (2023). Cryptanalysis of an image encryption algorithm based on two-dimensional hyperchaotic map. Entropy.

[52] Li C., Li S., Chen G., Halang W.A. (2009). Cryptanalysis of an image encryption scheme based on a compound chaotic sequence. Image Vis. Comput..

[53] Bakhshandeh A., Eslami Z. (2013). An authenticated image encryption scheme based on chaotic maps and memory cellular automata. Opt. Lasers Eng..

[54] Shakiba A. (2021). A randomized CPA-secure asymmetric-key chaotic color image encryption scheme based on the Chebyshev mappings and one-time pad. J. King Saud Univ. Comput. Inf. Sci..

[55] Li S., Chen Y., Chen L., Liao J., Kuang C., Li K., Liang W., Xiong N. (2023). Post-quantum security: Opportunities and challenges. Sensors.

[56] Gao J., Teng L. (2025). A chaotic multi-image encryption scheme based on block space jump scrambling and dynamic sliding queue diffusion. Nonlinear Dyn..

[57] Proos J., Zalka C. (2003). Shor’s discrete logarithm quantum algorithm for elliptic curves. Quantum Inf. Comput..

[58] Boudgoust K., Jeudy C., Roux-Langlois A., Wen W. (2022). On the Hardness of Module Learning with Errors with Short Distributions. J. Cryptol..

[59] Krawczyk H., Eronen P. (2010). HMAC-Based Extract-and-Expand Key Derivation Function (HKDF).

[60] USC-SIPI Image Database. Signal and Image Processing Institute, University of Southern California. https://sipi.usc.edu/database/.

[61] Ghorbani A., Saberikamarposhti M., Yadollahi M. (2022). Using ribonucleic acid (RNA) and Hénon map in new image encryption scheme. Optik.

[62] Shah A.A. (2020). Efficient image encryption scheme based on generalized logistic map for real time image processing. J. Real-Time Image Process..

[63] Deb S., Bhuyan B. (2021). Chaos-based medical image encryption scheme using special nonlinear filtering function based LFSR. Multimed. Tools Appl..

[64] Kumari P., Mondal B. (2023). Lightweight image encryption algorithm using NLFSR and CBC mode. J. Supercomput..

[65] Zhang X., Liu M., Yang X. (2023). Color image encryption algorithm based on cross-spiral transformation and zone diffusion. Mathematics.

